# Native Oxidation and Complex Magnetic Anisotropy‐Dominated Soft Magnetic CoCrFeNi‐Based High‐Entropy Alloy Thin Films

**DOI:** 10.1002/advs.202203139

**Published:** 2022-10-06

**Authors:** Junyi Zhang, Xiao Wang, Xiaona Li, Yuehong Zheng, Renwei Liu, Junhua Luan, Zengbao Jiao, Chuang Dong, Peter K. Liaw

**Affiliations:** ^1^ Key Laboratory of Materials Modification by Laser Ion and Electron Beams (Ministry of Education) School of Materials Science and Engineering Dalian University of Technology Dalian 116024 China; ^2^ State Key Laboratory of Advanced Processing and Recycling of Nonferrous Metals Lanzhou University of Technology Lanzhou 730050 China; ^3^ Shimadzu China Co. LTD Shanghai 200233 China; ^4^ Department of Materials Science and Engineering City University of Hong Kong Hong Kong 999077 China; ^5^ Department of Mechanical Engineering The Hong Kong Polytechnic University Hong Kong 999077 China; ^6^ Department of Materials Science and Engineering The University of Tennessee Knoxville TN 37996 USA

**Keywords:** electrical resistivity, high‐entropy thin films, magnetic anisotropic, native oxidation, soft magnetic

## Abstract

Soft magnetic high‐entropy alloy thin films (HEATFs) exhibit remarkable freedom of material‐structure design and physical‐property tailoring, as well as, high cut‐off frequencies and outstanding electrical resistivities, making them potential candidates for high‐frequency magnetic devices. In this study, a CoCrFeNi film with excellent soft magnetic properties is developed by forming a novel core–shell structure via native oxidation, with ferromagnetic elements Fe, Co, and Ni as the core and the Cr oxide as the shell layer. The core–shell structure enables a high saturation magnetization, enhances the electrical resistivity, and thus reduces the eddy‐current loss. For further optimizing the soft magnetic properties, O is deliberately introduced into the HEATFs, and the O‐incorporated HEATFs exhibit an electrical resistivity of 237 µΩ·cm, a saturation magnetization of 535 emu cm^−3^, and a coercivity of 23 A m^−1^. The factors that determine the ferromagnetism and coercivity of the CoCrFeNi‐based HEATFs are examined in detail by evaluating the microstructures, magnetic domains, chemical valency, and 3D microscopic compositional distributions of the prepared films. These results are anticipated to provide insights into the magnetic behaviors of soft magnetic HEATFs, as well as aid in the construction of a promising material‐design strategy for these unique materials.

## Graphical abstract

The CoCrFeNi film with excellent soft magnetic properties and high electrical resistivity is developed by forming a novel core–shell structure via native oxidation, with ferromagnetic elements Fe, Co, and Ni as the core and the Cr oxide as the shell layer. The Cr oxide weakens antiferromagnetic exchange interaction and increases the concentrations of ferromagnetic elements in the core.

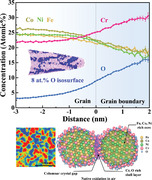

## 1 Introduction

Traditional soft magnetic bulk alloys are unsuitable for high‐frequency applications, because of their low magnetic conductivity at gigahertz (GHz) frequencies band and large eddy‐current losses.^[^ ^1^ ^]^ In comparison, soft magnetic thin films possess unique advantages of low magnetic losses, high cutoff frequencies, and permeability that originate from their remarkable electrical resistivities (*ρ*) and moderate anisotropic fields.^[^ ^2^, ^3^ ^]^ Further, soft magnetic high‐entropy alloy thin films (HEATFs) exhibit electrical resistivities that are superior to those of the other soft magnetic thin films. For instance, the *ρ* value of the Co_65_Ni_12_Fe_23_ film is only 21 µΩ·cm,^[^ ^4^ ^]^ while that of the Al_0.7_CoCrFeNi film can reach up to 540 µΩ·cm.^[^ ^5^ ^]^ Notably, high‐entropy alloys (HEAs) and HEATFs exhibit remarkable thermal stabilities, corrosion resistances, and robust mechanical properties.^[^ ^6^ *^–^* ^20^ ^]^ Thus, HEATFs are expected to broaden the composition range of the existing soft magnetic films.

The excellent soft magnetic properties of HEATFs are a recent discovery; for instance, the Fe_22_Mn_22_Co_27_Cr_21_Al_8_ film is characterized by a saturation magnetization (*M*_S_) of 80 emu g^−1^ and coercivity (*H*_C_) of 6.3 mT at room temperature (RT);^[^ ^21^ ^]^ the *M*_S_ for an electrodeposited CoCuFeNiZn film is 82 emu g^−1^, whereas the corresponding *H*_C_ and remanent magnetization (*M*_r_) are 19.5 Oe and 1.17%, respectively.^[^ ^22^ ^]^ Microwave devices fabricated using Fe_40_Co_35_Ni_5_Al_5_Cr_5_Si_10_ films (*M*_S_ = 9.13 × 10^5^ A m^−1^; *H*_C_ = 79.6 A m^−1^; and *ρ* = 375 µΩ·cm) have reportedly shown an improved quality factor (*Q*) relative to those of air‐core inductors.^[^ ^23^ ^]^ Furthermore, some researchers have studied the soft magnetic properties of high‐entropy oxide films. Mayandi et al. deposited a series of (CoCrCuFeNi)O*_x_* (*x* = 0.1–1.3) films by adding O during sputtering.^[^ ^24^ ^]^ These films exhibited micro‐segregation, leading to the formation of (Cr, O)‐rich, Cu‐rich, and (Fe, Co)‐rich phases that were alternately and uniformly distributed in the film. The O content changed the volume fractions in these regions, which affected the magnetic properties of the film. Minouei et al. fabricated high‐entropy (CrMnFeCoNi)_3_O_4_ thin films by adding O during annealing.^[^ ^25^ ^]^ The Curie temperature (*T*_C_) of the film reached up to ≈873 K, and the film exhibited good thermal stability, which is advantageous for sensors, spintronic devices, and magneto‐optical recording. However, annealing produced a large number of holes and pores in the films. These previous works demonstrated that the ferromagnetic properties of HEATFs could be tuned by partial oxidation via additions of O, which is also helpful for improving the mechanical properties of these films. Lee et al. revealed that the CoCrFeNi film could be reinforced, and its hardness could be increased by ≈14% through the in situ formation of Cr_2_O_3_ nanoparticle oxides.^[^ ^26^ ^]^

Currently, theoretical investigation results are mainly available for the bulk soft magnetic HEAs, whose magnetic properties have been found to be sensitive to their compositions and phase structures. The medium‐entropy alloy, CoFeNi, with a single‐phase face‐centered‐cubic (FCC) structure, exhibits ferromagnetic behavior at RT, with a maximum *M*_S_ of 1.671 T,^[^ ^27^ ^]^ while the single‐phase FCC CoCrFeNi HEA was paramagnetic at RT.^[^ ^28^, ^29^ ^]^ This difference in the magnetic behavior could be associated with the antiferromagnetic spin arrangement of Cr.^[^ ^30^ ^]^ Strong repulsion between the Cr atoms could induce the formation of a structure that exhibits a magneto‐resistive effect. In such a structure, the antiferromagnetic Cr atoms are located at the center, and the ferromagnetic elements form the first‐neighbor shell, resulting in weak magnetic, or even paramagnetic, properties.^[^ ^31^ ^]^ The ferromagnetic behavior becomes more significant with the appearance of the body‐centered cubic (BCC) phase. For example, Al addition promotes the formation of BCC phases in the CoCrFeNi HEA, and the resulting Al_1.25_CoCrFeNi HEA exhibits an *M*_S_ of 301.35 emu g^−1^.^[^ ^32^ ^]^ The *H*_C_ of the HEAs is dominated by the movement of domain walls and irreversible changes in domain rotation, which are sensitive to microstructure, impurities, deformation, grain sizes, stress, and heat treatment.^[^ ^33^, ^34^ ^]^ The equiaxial crystal structure changes into a columnar crystal structure during the directional solidification of the FeCoNiAl_0.2_Si_0.2_ alloy, whose *H*_C_ is reduced from 1400 to 315 A m^−1^ owing to the presence of fewer grain‐boundary defects.^[^ ^35^ ^]^ In the case of the Al_1.5_Co_4_Fe_2_Cr alloy, the *H*_C_ decreases to 127 A m^−1^ because of the uniform distribution of the BCC ferromagnetic nano‐precipitates in the B2 matrix phase.^[^ ^36^ ^]^ After the rapid solidification of the FeCoNiAl_0.2_Si_0.2_ alloy, the increasing microstructural homogeneity reduces the *H*_C_ from 406 to 86 A m^−1^.^[^ ^37^ ^]^

However, the theoretically derived results of the bulk HEAs cannot be directly applied to the HEATFs owing to the differences in coordination numbers, lattice constants (especially at a direction perpendicular to the surface), and valence electron (3d, 4s) distributions. Furthermore, the residual stress generated during the fabrication of HEATFs affects their magnetic properties.^[^ ^38^, ^39^ ^]^ Therefore, detailed investigations are required to reveal the magnetic properties of HEATFs.

In this study, CoCrFeNi‐based HEATFs were prepared by radio frequency (RF) magnetron sputtering, and they showed excellent soft magnetic properties through native oxidation. Further, the factors affecting the soft magnetism were identified and analyzed. The ferromagnetism was found to be related to the native oxidation and lattice distortion, and the coercivity was associated with the complex magnetic anisotropy and magnetic domain sizes. More significantly, in the core–shell structure of the CoCrFeNi_thick_ film, the ferromagnetic FeCoNi formed the matrix phase, and Cr oxide was the weak‐magnetic high‐resistance coated layer, which improved the electrical resistivity and reduced the eddy‐current losses. The core–shell structure improved the high‐frequency soft magnetic properties of the HEATFs. The addition of Al changed the core–shell structure into a composite structure with Fe, Co, Ni, and Cr in the core, and Al oxide in the shell. This structural modification would induce a slightly decrement in the soft magnetism but increase the electrical resistivity, broadening the comprehensive performance range of the films. Moreover, O was added intentionally in the CoCrFeNi oxide thin films to further improve both the soft magnetism and electrical resistivity to explore their potential applications in various fields. The series of CoCrFeNi‐based HEATFs fabricated in this study can be employed to develop soft magnetic electronic devices because of their large composition‐ and performance‐modulation ranges.

## 2 Experimental Section

### 2.1 Material Preparation

A series of CoCrFeNi_thin_, CoCrFeNi_thick_, and Al*_x_* CoCrFeNi (*x* = 0.1–0.5) HEATFs were deposited on single‐crystal Si (100) substrates using a JGP450 radio frequency (RF) magnetron‐sputtering system. An equiatomic Co—Cr—Fe—Ni quaternary sputtering target (diameter: 75 mm) was prepared by arc‐melting in an Ar atmosphere. The five‐element alloy combination target was designed through the adhesion of Al pieces (*Φ*8 mm × 1 mm) in the sputtering areas of the CoCrFeNi target. The purity of each component was not less than 99.9 wt%. HEATFs with different compositions were obtained by changing the number of Al pieces (1–5) during the sputtering process. The background vacuum was less than 3.0 × 10^−4^ Pa, and the temperature of the substrate was maintained at ≤323 K. The substrate rotated at a constant speed of 10 r min^−1^, and the working distance was ≈10 cm. Ar gas [the purity is 99.999 volume percent (vol%)] was filled to a pressure of ≈1.4 Pa to build up luminance, and the flow rate was set to 30 standard cubic centimeter per minute (sccm) during the sputtering. The pre‐sputtering time was 40 min, followed by 60 min of formal sputtering. A thick quaternary film was prepared under a 110 W RF power supply, while the other films were fabricated under a 100 W RF power supply without bias.

Two types of high‐entropy oxide films, namely, CoCrFeNiO_0.4_ and CoCrFeNiO_0.8_ films, were prepared using the same magnetron sputtering system with a sputtering power of 100 W, working pressure of 0.3 Pa, formal sputtering time of 90 min, and a total O_2_/Ar mixture gas (99.999 vol% purity) flow rate of 30 sccm. The O content was adjusted by changing the O_2_/Ar ratio in the gas mixture.

All the sputtering‐synthesized films were divided into three groups. The actual compositions (electron probe micro‐analyzer (EPMA) measurements) and thicknesses (cross‐sectional scanning electron microscopy (SEM) measurements) of the HEATFs are shown in **Table** **1**. The concentrations of the four metal components (Co, Cr, Fe, and Ni) were close to the equal molar ratio, although a slight compositional fluctuation in each element of the films due to the different sputtering thresholds and rates of these elements. The two Al‐free films belonging to Group 1 were found to be 378 and 608 nm thick and were referred to as CoCrFeNi_thin_ and CoCrFeNi_thick_, respectively. The other films, categorized in Group 2, contained Al in the range of 2.11–10.53 at%, were 335 to 369 nm thick, and were named as Al*_x_* CoCrFeNi (*x* = 0.1–0.5, atomic ratios). The two films of Group 3 contained 8.54 and 16.99 at% O, were 690 and 814 nm thick, and were called as CoCrFeNiO*_x_* (*x* = 0.4 and 0.8, atomic ratios). O was added to optimize the soft‐magnetic performance of the HEATFs.

**Table 1:** Film compositions measured by EPMA and film thicknesses measured by SEM cross‐section analysis for CoCrFeNi_thin_, CoCrFeNi_thick_, Al*_x_* CoCrFeNi (*x* = 0.1–0.5, atomic ratios), and CoCrFeNiO*_x_* (*x* = 0.4 and 0.8, atomic ratios) films

Groups No.	Composition [at%]						Film thickness [nm]	Naming of the films
	Al	Co	Cr	Fe	Ni	O		
1	‐	24.58	24.44	21.52	29.46	‐	378	CoCrFeNi_thin_
	‐	26.78	24.55	23.97	24.70	‐	608	CoCrFeNi_thick_
2	2.11	23.72	23.62	21.35	29.20	‐	369	Al_0.1_CoCrFeNi
	3.78	23.61	23.26	20.89	28.46	‐	360	Al_0.2_CoCrFeNi
	6.51	22.01	22.37	20.58	28.53	‐	352	Al_0.3_CoCrFeNi
	8.16	22.30	22.03	19.85	27.66	‐	368	Al_0.4_CoCrFeNi
	10.53	21.86	21.35	19.15	27.11	‐	335	Al_0.5_CoCrFeNi
3	‐	24.49	22.49	21.16	23.32	8.54	690	CoCrFeNiO_0.4_
	‐	22.38	20.26	19.16	21.21	16.99	814	CoCrFeNiO_0.8_

### 2.2 Material Characterizations

The surface morphologies and thicknesses of the prepared HEATFs were analyzed by SEM (Zeiss Supra55, Baden‐Wurttemberg, Germany). The corresponding crystal structures were investigated by Grazing‐angle incidence X‐ray diffraction (Empyrean, PANalytical B.V., Netherlands), and transmission electron microscopy (TEM, JEM 2100F, JEOL, Japan) was employed to characterize the microstructures and morphologies of the films. The TEM specimens were fabricated by mechanical grinding and precision ion polishing (PIPS, Gatan 695, Pleasanton, USA). The film curvatures were identified using a 3D Optical Surface Profiler (OSP, NewView 9000, ZYGO, USA).

The chemical compositions of the HEATFs were analyzed by a wavelength dispersive spectrometer equipped with an EPMA (EPMA‐1600, Shimadzu, Japan), and an energy‐dispersive X‐ray spectroscopy (EDS) equipped with TEM (EDS‐TEM). The 3D atomic distributions of the HEATFs were evaluated using local electrode atom probe tomography (APT, LEAP 5000 XR, CAMEACA, France). Needle‐shaped specimens required for the APT were fabricated by a focused ion beam/scanning electron microscope (FIB/SEM, Scios, FEI, USA). The workstations AP Suite 6.1 was used for the 3D reconstructions and data analyses.

The chemical states were analyzed by X‐ray photoelectron spectroscopy (XPS, EscaLab XI+, thermos, UK). A four‐point probe tester (RTS‐9, 4 Probes Tech, Guangzhou, China) was used to measure the RT electrical resistivity of the HEATFs. The hysteresis loops were measured by a vibrating sample magnetometer (Lakeshore‐7400S, Columbus, USA). The magnetic domain structures were determined using a scanning probe microscope operated in the magnetic force mode (SPM, SPM‐9700HT, Shimadzu, Japan). The lifting distance of the probe was >100 nm, and the measuring range was 1 µm × 1 µm.

## 3 Results

### 3.1 Microstructural Characterization

**Figure 1:**
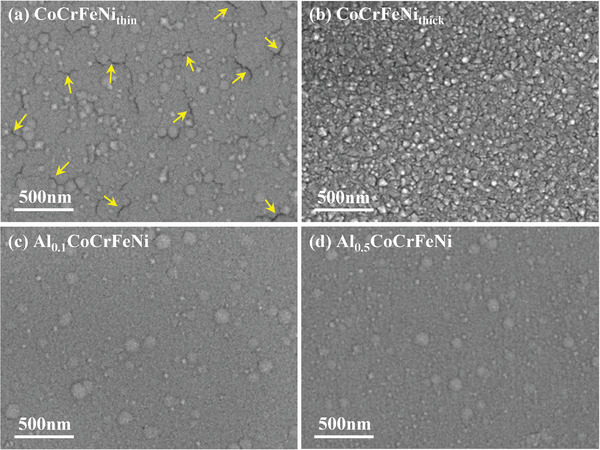
The SEM secondary‐electron images of the surface morphologies of a) CoCrFeNi_thin_, b) CoCrFeNi_thick_, c) Al_0.1_CoCrFeNi, and d) Al_0.5_CoCrFeNi HEATFs.

**Figure** **1** shows the SEM secondary‐electron images of the surface morphologies exhibited by the CoCrFeNi_thin_, CoCrFeNi_thick_, Al_0.1_CoCrFeNi, and Al_0.5_CoCrFeNi HEATFs; they all exhibit columnar crystal clusters (the characterization results of other films are presented in Figure S1, Supporting Information). A small number of microcracks, marked by arrows in Figure 1a, are distributed on the surface of the CoCrFeNi_thin_ film, while the surface morphology of the CoCrFeNi_thick_ film exhibits irregularly shaped particles without microcracks. To understand this phenomenon, a white light interferometer was used to determine the average curvature radii in different directions as well as the profiles of the CoCrFeNi_thin_ and CoCrFeNi_thick_ films, as listed in **Table** **2**. Both the films exhibited spherical concave surfaces because of the strong residual stress induced by the lattice mismatch and differences in the film/substrate thermal expansion coefficients. The stress difference was compared using the Stoney equation:^[^ ^40^ ^]^

(1) $$\sigma_{\mathrm{films}}=\frac{E}{6\left(1-\nu \right)}\;\frac{t_{s}^{2}}{t_{f}}\frac{1}{R}$$

where *σ*_films_ is the average stress in the film; *E* and *ν* are the Young's modulus and Poisson's ratio of the Si(100) substrate (171 GPa and 0.28, respectively); *t*_s_ is the thickness of the substrate (0.5 mm); *t*_f_ is the thickness of the film; and *R* is the change in the radius of curvature of the substrate because of film deposition (the Si substrate was a smooth and flat surface after the directional cutting and precision polishing, and thus, the change in the curvature of the sample surface after the deposition was approximately equal to the film curvature).

**Table 2:** Measured average curvature radii, calculated stresses of X and Y directions, and the profiles of the CoCrFeNi_thin_ and CoCrFeNi_thick_ films

Film	*t*_f_ [nm]	*R*_X_ [m]	*R*_Y_ [m]	*σ*_X_ [GPa]	*σ*_Y_ [GPa]	Profile
CoCrFeNi_thin_	378	52.863	33.584	0.376	0.593	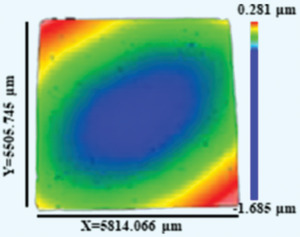
CoCrFeNi_thick_	608	46.577	24.971	0.266	0.496	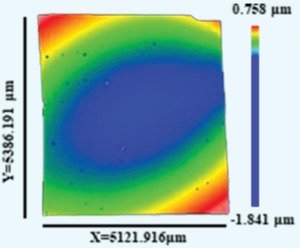

The stress values in the X and Y directions for the CoCrFeNi_thin_ and CoCrFeNi_thick_ films are listed in Table 2. Although the microcracks in the CoCrFeNi_thin_ film partially released the residual stress,^[^ ^41^ ^]^ a more significant stress still remained in both the directions, compared with that of CoCrFeNi_thick_ film, which also was the primary source of microcrack generation in the CoCrFeNi_thin_ film.

**Figure 2:**
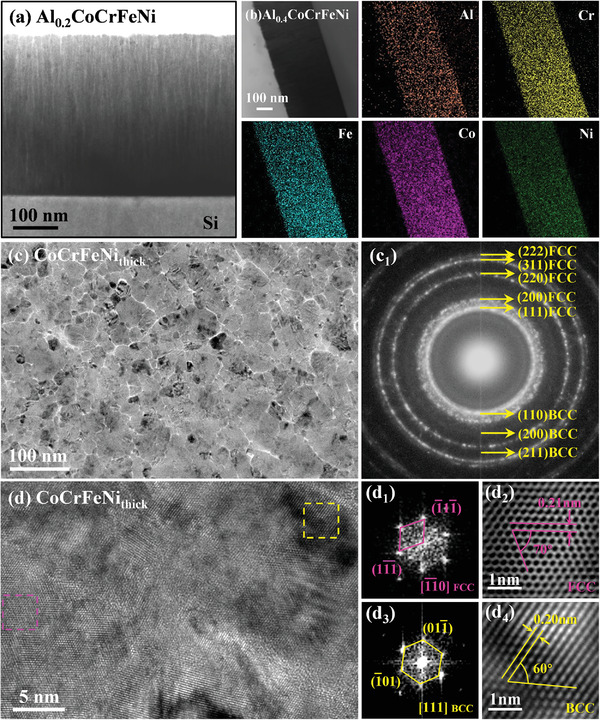
a) Cross‐sectional TEM bright‐field image of the Al_0.2_CoCrFeNi film. b) The cross‐sectional STEM‐HAADF image of the Al_0.4_CoCrFeNi film and the corresponding EDS mappings of each elements in the same area. c) The planar TEM bright‐field image of the CoCrFeNi_thick_ film and c_1_) the corresponding SAED pattern. d) The HRTEM images, d_1_,d_3_) the corresponding FFT spectra and d_2_,d_4_) filtered images of the FCC and BCC structures of the CoCrFeNi_thick_ film.

The microstructures of all the HEATFs were composed of columnar nanocrystals, suggesting that the films exhibited a clear preferred orientation during the growth process, as evident from the cross‐sectional TEM bright‐field image of the Al_0.2_CoCrFeNi film displayed in **Figure** **2**a. This image also shows that the films have a smooth film‐substrate interface with uniform thickness. Figure 2b presents the cross‐sectional scanning/transmission electron microscopy high‐angle annular dark‐field (STEM‐HAADF) image and the corresponding EDS mapping analysis of the Al_0.4_CoCrFeNi film. These results indicate that the elements Al, Co, Cr, Fe, and Ni distribute randomly in the film. For a more detailed analysis, the composition distribution was investigated on smaller scales, and the corresponding results are described in subsequent sections.

Figure 2c presents the planar TEM bright‐field image of the CoCrFeNi_thick_ film, indicating grain sizes of ≈17.6 ± 0.7 nm. The corresponding selected‐area electron diffraction (SAED) pattern shown in Figure 2c_1_ confirms the presence of the FCC and BCC phases in the films. Moreover, the weakly diffraction rings of the BCC phase are indicative of the dominant role of the FCC phase in the films. This observation was confirmed by analyzing the high‐resolution TEM images, the corresponding fast Fourier transform (FFT) spectra, and the filtered images of the CoCrFeNi_thick_ film shown in Figure 2d. Additionally, the average grain size of the CoCrFeNi_thick_ film was larger than that of the CoCrFeNi_thin_ film, while those of the Al*_x_* CoCrFeNi films were essentially the same (other results are shown in Figures S2–S4, Supporting Information).

According to previous studies, the microstructures of the as‐cast bulk CoCrFeNi alloys prepared by conventional smelting are composed of a single FCC phase.^[^ ^42^, ^43^, ^44^ ^]^ Because of the addition of Al into the CoCrFeNi alloys, the resulting Al*_x_* CoCrFeNi exhibit FCC and BCC dual‐phases at *x* > 0.5.^[^ ^43^ ^]^ However, the microstructures of the CoCrFeNi_thin_ and CoCrFeNi_thick_ films prepared in this study were already composed of the FCC and BCC dual‐phases, and the content of the BCC phase in the Al*_x_* CoCrFeNi films increased with the increasing Al concentration, although *x* was less than 0.5. The magnetron‐sputtering process is a non‐equilibrium preparation method with a drastic cooling rate (≈10^9^ K s^−1^).^[^ ^45^ ^]^ Ichikawa et al. indicated that higher cooling rates result in a higher degree of undercooling (Δ*T*).^[^ ^46^ ^]^ Therefore, magnetron sputtering can result in an extreme Δ*T* because of the lower diffusivity of the atoms and the retardation of the grain nucleation. The BCC phase would form when there is a large Δ*T* in the CoCrFeNi alloy, as corroborated by An et al. via thermo‐dynamic calculations.^[^ ^47^ ^]^ In addition, several researchers also found that the FCC and BCC dual‐phased structure of the CoCrFeNi alloy is closely related to the large Δ*T*,^[^ ^48^, ^49^, ^50^ ^]^ with severe lattice distortion.^[^ ^48^ ^]^ These previously reported findings demonstrate that an FCC and BCC dual‐phased structure can be obtained in the CoCrFeNi film during the magnetron sputtering.

**Figure 3:**
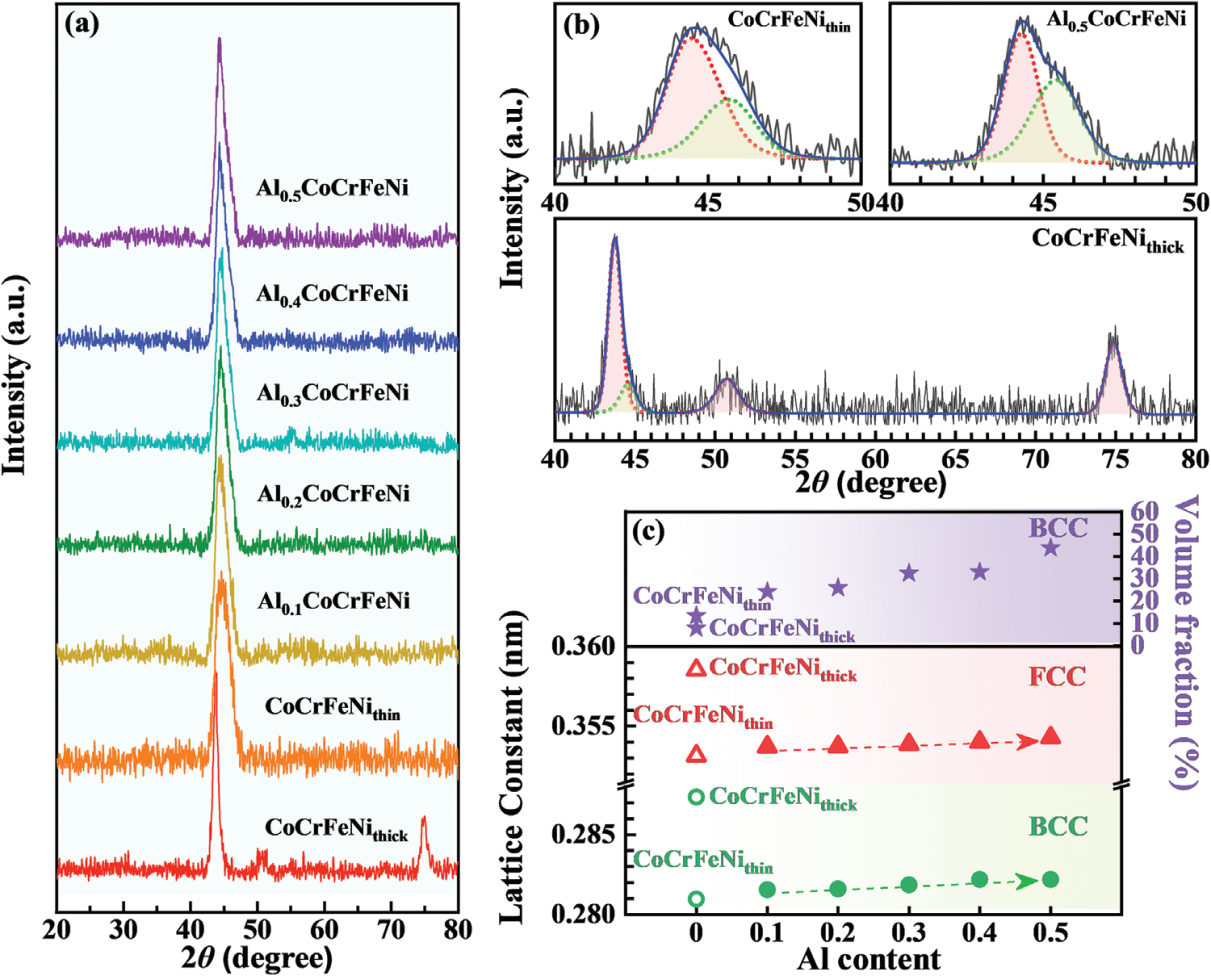
a) XRD patterns and b) fitting results of the diffraction peak of the HEATFs (red and green dash lines correlate to the diffraction peaks of FCC and BCC phases; the blue solid line is the sum of the fitting curves). c) Lattice constant and BCC phases volume fraction of the HEATFs as functions of the Al content and thickness.

The XRD patterns of the HEATFs are depicted in **Figure** **3**a, indicating three obvious diffraction peaks (43.75°, 50.73°, and 74.88°) for the CoCrFeNi_thick_ film. In contrast, the spectra of the other films show only one wide diffraction peak in the range of 40°–50°. The two peaks located at 50.73° and 74.88° correspond to the {200}_FCC_ and {220}_FCC_ phase, respectively, while the wide peak located at 40°–50° can be attributed to the overlapping of the peaks corresponding to the {111}_FCC_ and {110}_BCC_ phases according to the results of the previously reported investigations on bulk HEAs or HEATFs with similar compositions.^[^ ^13^, ^51^ ^]^ To determine the lattice constants and contents of the two phases in the HEATFs, peak separation and fitting were performed using the JADE software, and the peak intensities were normalized.^[^ ^52^, ^53^ ^]^

Figure 3b shows the fitting results of the XRD patterns obtained for the CoCrFeNi_thin_, CoCrFeNi_thick_, and Al_0.5_CoCrFeNi films (the fitting results of the other films are presented in Figure S5, Supporting Information). Compared with the spectrum of the CoCrFeNi_thick_ film, that of the CoCrFeNi_thin_ film shows right‐shifting of the diffraction peaks corresponding to {111} _FCC_ and {110} _BCC_ phases. Figure 3c displays the lattice parameters (*a*) of the FCC and BCC phases in the films, these parameters were determined by full spectrum fitting. The lattice parameters corresponding to the FCC and BCC phases in the CoCrFeNi_thin_ film were 0.352 and 0.281 nm, respectively, while those of the CoCrFeNi_thick_ film were 0.358 and 0.285 nm, respectively.

With the increasing Al content, the diffraction peaks corresponding to the FCC and BCC phases gradually shifted to the left, indicating that the lattice parameters of the two phases increased continuously, from 0.353 to 0.355 nm and from 0.281 to 0.283 nm, respectively. This increase in the lattice parameters can be ascribed to the atomic radius of Al, which is 15% larger than the average radius of the other components.^[^ ^54^ ^]^ Moreover, the diffraction peak intensity is closely related to the volume fraction of phases. Therefore, the relative intensity ratios of the FCC and BCC phases in the XRD patterns of the CoCrFeNi_thick_, CoCrFeNi_thin_, and Al*_x_* CoCrFeNi (*x* = 0.1–0.5) films, can be used to determine the volume fraction of the BCC phases (details in the Supporting Information), and the calculated results are shown in Figure 3c. It indicates that CoCrFeNi_thick_ film had the lowest BCC content, and the value increases with Al addition (Al promotes phase transformation from FCC to BCC).^[^ ^55^, ^56^ ^]^

### 3.2 Room‐Temperature Electrical Resistivity

**Figure 4:**
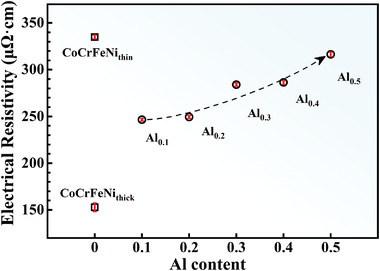
The room temperature (RT) electrical resistivity (*ρ*) variation with the Al content and thickness of HEATFs.

As shown in **Figure** **4**, the RT electrical resistivity (*ρ*) of the CoCrFeNi_thin_ film was 335.0 ± 2.09 µΩ·cm, while that of the CoCrFeNi_thick_ film dropped significantly to 153.0 ± 4.52 µΩ·cm due to its higher thickness. The *ρ* values of the Al*_x_* CoCrFeNi films monotonically increased from 246.5 ± 2.29 to 316.4 ± 2.39 µΩ·cm with the increasing Al content. Overall, the *ρ* values of the films were larger than 150 µΩ·cm, indicating that these films are high‐resistance materials.^[^ ^57^ ^]^

The source of the high *ρ* for the CoCrFeNi‐based HEATFs was discussed in our previous report, including the following four aspects:^[^ ^5^ ^]^ 1) Severe lattice distortion caused by the chemical disorder and increased residual stress (film/substrate lattice mismatch); 2) Increased electron scattering owing to substantial grain boundaries in the films with columnar nanocrystals; 3) s‐d scattering effects in transition metals; and 4) local spin interactions of the ferromagnetic Fe, Co, and Ni atoms.

The *ρ* of the CoCrFeNi_thin_ film was significantly higher than that of the CoCrFeNi_thick_ film, and this difference in the *ρ* value can be attributed to the increased probability of electron scattering and decreased mean free path of the electrons caused by the increased number of grain boundaries and microcracks in the CoCrFeNi_thin_ film. In the Al*_x_* CoCrFeNi films, the s‐d scattering and magnetic spin‐scattering effects become weak with the increasing Al content. However, their *ρ* still increases because of the more severe lattice distortion.

### 3.3 Soft Magnetic Properties of the High‐Entropy Alloy Thin Films

**Figure 5:**
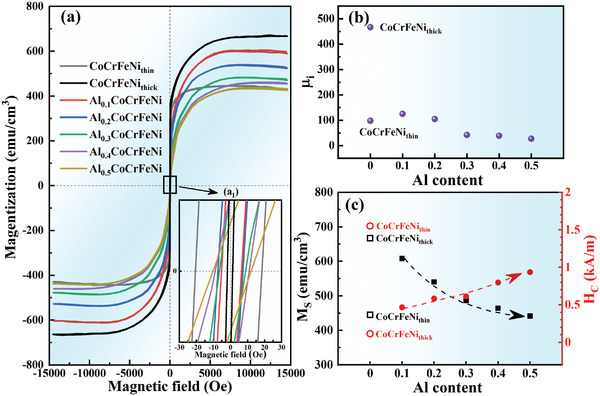
a) In‐plane (parallel) magnetic‐hysteresis loops of HEATFs at RT, b) *µ*_i_, and c) *M*_S_, *H*_C_ variation with the Al content and thickness of HEATFs.

The in‐plane magnetic hysteresis loops of the HEATFs, with the enlarged images, are shown in **Figure** **5**a,a_1_. All the films reached saturation magnetization under a weak applied magnetic field without significant hysteresis effects, indicating soft magnetic properties with a high initial permeability (*µ*_i_) and *M*_S_, as well as a low *H*_C_, which less than 20 Oe (1.572 kA m^−1^).

The variation in the average value of the magnetization (*M*_avg_) with the external magnetic field, *H*, can be derived from the magnetic‐hysteresis loops. *M*_avg_ can be expressed by the following equation:^[^ ^58^ ^]^

(2) $$M_{\mathrm{avg}}=\frac{M_{u}+M_{e}}{2}$$

where *M*_u_ and *M*_e_ are the magnetization values of the upper and lower halves of the hysteresis loop in the first and fourth quadrants, respectively. Then, the *M*_avg_ versus *H* curves were fitted by the method in the fitting section of Supporting Information.

Figure 5b shows the values of *µ*_i_ for the HEATFs, calculated using the following equation:^[^ ^38^, ^39^ ^]^

(3) $$\mu_{i}=\lim_{H\to 0}\;\left(1+M_{\mathrm{avg}}/H\right)$$

According to these calculations, all the films exhibited ferromagnetic behavior. The *µ*_i_ of the CoCrFeNi_thick_ film (*µ*_i_ = 466) was the highest, while that of the Al*_x_* CoCrFeNi films decreased to 27 with the increasing Al concentration.

The CoCrFeNi_thick_ film exhibited the highest *M*_S_ and the lowest *H*_C_ (*M*_S_ = 667 emu cm^−3^, *H*_C_ = 0.12 kA m^−1^), while the CoCrFeNi_thin_ film showed the opposite trend, that is, the lowest *M*_S_ and the highest *H*_C_ (*M*_S_ = 445 emu cm^−3^, *H*_C_ = 1.55 kA m^−1^), as depicted in Figure 5c. With the increasing Al content, the *M*_S_ decreased from 638 to 441 emu cm^−3^, and the *H*_C_ increased from 0.47 to 0.94 kA m^−1^, indicating that a variation in the film thickness produces a more significant effect on the *M*_S_ and *H*_C_.

**Figure 6:**
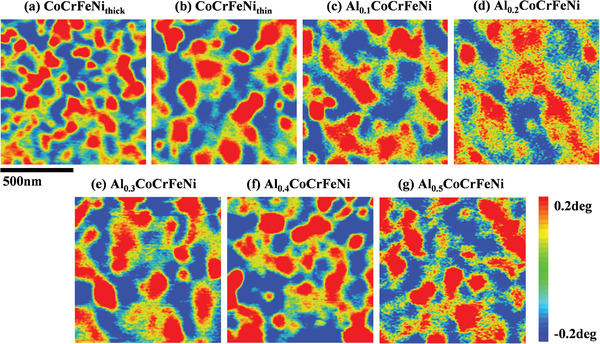
The magnetic domain structure of HEATFs under magnetic force mode in the SPM measurement.

**Figure** **6** depicts the magnetic domain structures of the HEATFs. All the films had maze‐like closed magnetic domains with rounded or elongated regions, and the domain sizes were larger than the average grain sizes. The CoCrFeNi_thick_ film exhibited the smallest magnetic domain size (≈65 nm), while those of the other films showed no difference (≈90 nm). This indicates that the magnetization directions of several adjacent crystal grains in the HEATFs are aligned in the same direction. The maze‐like closed magnetic domains are induced by the reduced magnetostatic energy and increased total Bloch wall area related to the vertical anisotropy, such domains often appear in the films with columnar nanocrystals.^[^ ^59^, ^60^ ^]^

### 3.4 Native Oxidation State Characterization

**Figure 7:**
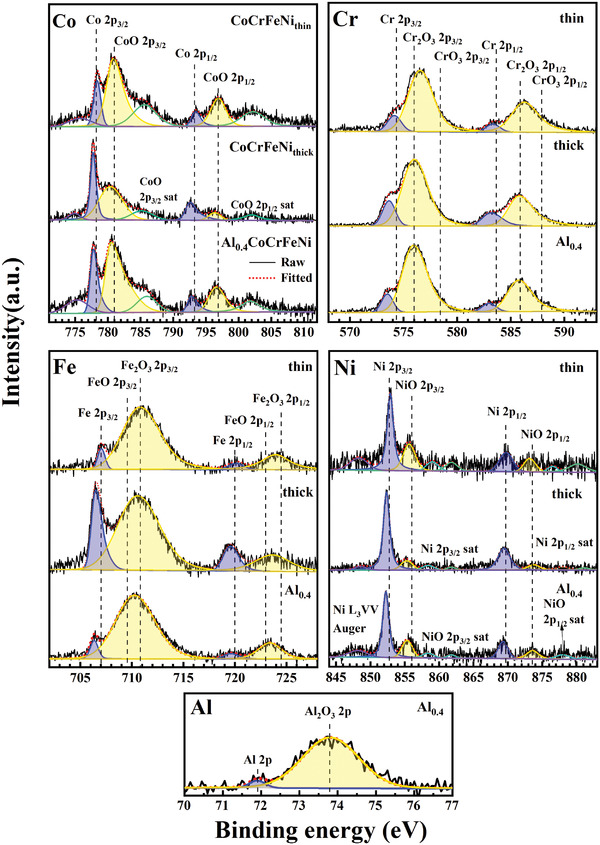
Single‐elemental XPS analyses of CoCrFeNi_thin_, CoCrFeNi_thick_, and Al_0.4_CoCrFeNi films.

The single‐elemental XPS spectra of the CoCrFeNi_thin_, CoCrFeNi_thick_, and Al_0.4_CoCrFeNi films surfaces are shown in **Figure** **7**. As evident, the surfaces were in the state of native oxidation, and therefore, the corresponding metal peaks (blue peak on the left) and oxidation peaks (yellow peak on the right) for each element can be observed. In addition, the green and purple peaks represent the satellite and Auger peaks of the corresponding elements, respectively. The black solid and red dashed lines represent the measured and fitting results, respectively.

According to the surface‐oxidation states of the Co, Cr, Fe, and Ni transition metals, the elements constituting the prepared films can be clearly divided into two categories: 1) All the Fe, Co, and Ni elements are all in their native oxidation states (Fe^3+^, Co^2+^, and Ni^2+^) in the CoCrFeNi_thin_ and CoCrFeNi_thick_ films. After adding Al, the oxidation peak position of these elements slightly shifts to lower binding energies, indicating a weaker degree of oxidation. 2) In the CoCrFeNi_thin_ and CoCrFeNi_thick_ films, the Cr valence state is between + 3 and + 6, which is higher than that of its native oxidation state (Cr^3+^), indicating aggravated oxidation of Cr in the films. However, in the Al_0.4_CoCrFeNi films, the severely oxidized Cr is replaced by Al and returns to its native‐oxidation state.

The surface‐oxidation state of each element is associated with its vacancy‐formation energy and electronegativity. Fe, Co, and Ni exhibit positive vacancy‐formation energies, whereas Cr shows a negative vacancy‐formation energy.^[^ ^61^ ^]^ Moreover, compared with Fe, Co, and Ni, Cr is weakly electronegative.^[^ ^62^ ^]^ Therefore, Cr is more oxyphilic than the other elements. The addition of Al to the CoCrFeNi thin films results in a more violent Al—O reaction than the Cr—O reaction, because the electronegativity of Al (1.61) is less than that of Cr (1.66),^[^ ^62^ ^]^ thus, Cr returns to its native‐oxidation state.

## 4 Discussion

### 4.1 Saturation Magnetization Analysis of High‐Entropy Alloy Thin Films Ferromagnetic Behavior

#### 4.1.1 Origin of the Ferromagnetic Behavior

As mentioned in the previous section, the FCC phases of the bulk CoCrFeNi‐based HEAs show paramagnetic behavior at RT, while the BCC phases are ferromagnetic. However, the CoCrFeNi HEATFs prepared in this study exhibited FCC phases with a small amount of BCC phase, which were all ferromagnetic.

Based on quantum mechanics, spontaneous magnetization in metals originates from electrostatic‐exchange effect between the electrons in the atoms or neighboring atoms. According to the Heisenberg exchange model:^[^ ^39^ ^]^

(4) $$E_{\mathrm{ex}}=-2\sum_{i<j}A_{ij}S_{i}\cdot S_{j}$$

where *E*_ex_ is the exchange energy between atoms, *A_ij_* is the exchange integral between the electrons of the atoms, *S_i_* and *S_j_* are the maximum total numbers of spins combined with the uncancelled electron spins in each atom. *A_ij_* > 0 shows ferromagnetism, and *A_ij_* < 0 indicates anti‐ferromagnetism.

**Figure 8:**
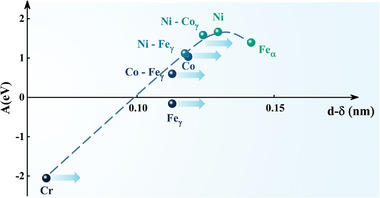
The relationship curve between the exchange energy (*A*) and (*d–δ*) given by Neel (*d* is the distance between atoms, and *δ* is the electron orbit radii of 3d or 4f).

The exchange integral (*A*) is related to the exchange interaction distance, *d–δ*, (see **Figure** **8**), as confirmed by Neel's theory;^[^ ^63^ ^]^ *d* represents the distance between two neighboring atoms, and *δ* is the total electron orbit radii (3d or 4f). For common metals or alloys with an FCC structure, the constituent Co—Co, Ni—Ni, Ni—Co, Ni—Fe, and Co—Fe pairs exhibit ferromagnetic exchange interactions (*A* > 0), while the Fe‐Fe pairs exhibit antiferromagnetic exchange interactions (*A* < 0). However, in metals or alloys with a BCC structure, the Fe—Fe pairs exhibit ferromagnetic exchange interactions (*A* > 0), while the Cr—Cr pairs undergo strong antiferromagnetic exchange interactions (*A* < 0).

Thus, a universal viewpoint is that the two exchange interactions (*A* > 0 and *A* < 0) can mutually offset in many bulk CoCrFeNi HEAs, which has an FCC structure, and as a result, these alloys usually exhibit a weakly magnetic or paramagnetic behavior. However, this is not absolute. In CoCrFeNi‐based HEAs with severe lattice distortion, the magnetic exchange distance changes greatly, resulting in that Fe—Fe pairs in the FCC phase show the ferromagnetic exchange interaction, while the Cr—Cr pairs in the BCC phase exhibit weak, or even negligible, antiferromagnetic exchange interactions, and in this case, the HEAs can exhibit ferromagnetism behavior.^[^ ^64^ ^]^ Therefore, the lattice distortion in the HEAs can affect the magnetic exchange distance, and thus, induce variations in the ferromagnetic behavior.

According to the aforementioned discussion, the ferromagnetism source can then be determined by considering the following three aspects in the current CoCrFeNi‐based HEATFs.

##### Reinforced Ferromagnetic Exchange Due to Severe Lattice Distortion

Lattice distortion is one of the HEAs’ characteristics that arises because of the lack of chemical periodicity and various sizes of the solute atoms.^[^ ^65^, ^66^ ^]^ The bulk alloys are prepared by the conventional melting methods, while the current HEATFs are fabricated by a non‐equilibrium method that induces more chemical disordering. Furthermore, a significant stress exists in the HEATFs because of the different film/substrate thermal‐expansion coefficients. Consequently, the lattice distortions exhibited by these films is more than that in bulk alloys.^[^ ^48^, ^67^ ^]^

The *d–δ* values of the FCC phase in the CoCrFeNi_thick_ film and those of the reported single‐phase FCC CoCrFeNi bulk alloy are presented in **Table** **3**. To calculate *d–δ*, the lattice constant, *a*_FCC_, of the CoCrFeNi_thick_ film was determined by fitting the XRD patterns (*a*_avg_ = 0.356 nm; *a*_max_ = 0.365 nm), and the *d*_avg_ value of the CoCrFeNi bulk HEA was extracted from the reported lattice constant of the FCC phase (*a*_FCC_ = 0.356 nm).^[^ ^68^ ^]^ In the CoCrFeNi_thick_ film, the *d*_avg_ and *d*_max_ are 0.254 and 0.258 nm, respectively. In contrast, the *d*_avg_ of the bulk alloy is only 0.252 nm, and the total of the 3d or 4f electron orbital radius (*δ*) of all the atom pairs is constant. Therefore, *d–δ* for all the atom pairs in films are enlarged relative to those of the bulk alloys. According to the trend of the dotted line in Figure 8, the *d–δ* values of the Cr—Cr and Fe—Fe pairs increases, and *A* approaches (from negative values) to 0, exhibiting a weakened antiferromagnetic exchange interaction. Moreover, the *d–δ* values of the Co—Co, Ni—Ni, Ni—Co, Co—Fe, and Ni—Fe pairs increase. *A* becomes larger in the positive range, and an enhanced ferromagnetic exchange interaction can be obtained. Therefore, the counteracting effects of the two exchange interactions reduce, and the films exhibit ferromagnetic behavior.

**Table 3:** The exchange interaction distance (*d*–*δ*) of the FCC phase for the CoCrFeNi_thick_ film and CoCrFeNi bulk alloy with a single‐phase FCC

Pairs	*δ* [nm]^[^ ^63^ ^]^	Bulk—CoCrFeNi^[^ ^68^ ^]^		CoCrFeNi_thick_			
		*d*_avg_ [nm]	(*d*–*δ*) _avg_ [nm]	*d*_avg_ [nm]	(*d*–*δ*) _avg_ [nm]	*d*_max_ [nm]	(*d*–*δ*) _max_ [nm]
Cr—Cr	0.181	0.252	0.071	0.254	0.073	0.258	0.077
Fe—Fe	0.144		0.108		0.110		0.114
Co—Co	0.130		0.122		0.124		0.128
Ni—Ni	0.119		0.133		0.135		0.139
Ni—Co	0.125		0.127		0.129		0.133
Co—Fe	0.137		0.115		0.117		0.121
Ni—Fe	0.130		0.122		0.124		0.128

##### Micro‐Segregation and Preferential Oxidation of Antiferromagnetic Cr

**Figure 9:**
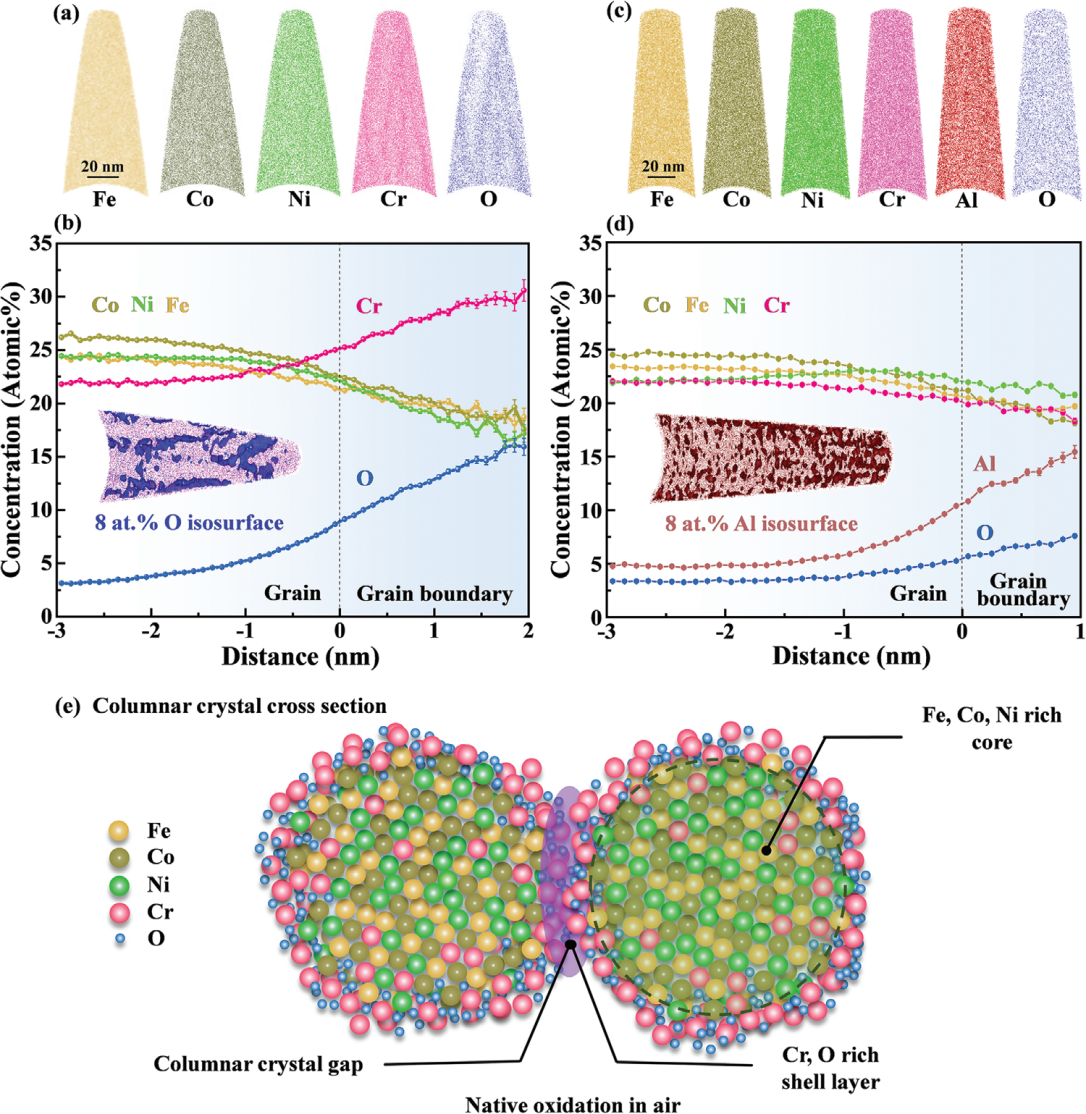
Atom probe analysis of the CoCrFeNi_thick_ and Al_0.4_CoCrFeNi HEATFs: a,c) elemental atom maps of Fe, Co, Ni, Cr, Al, and O, b,d) reconstruction maps with isosurfaces of the 8 at% O or 8 at% Al and the corresponding proximity histogram concentration profile showing the compositional partitioning of O (Al) rich and O (Al) poor zone. e) Schematic illustration of the core–shell structure of the CoCrFeNi_thick_ film.

There were many narrow columnar crystal gaps in the CoCrFeNi_thick_ film, and O atoms diffused to the film interior through these gaps. Accordingly, preferential oxidation of antiferromagnetic Cr occurs, inducing micro‐segregation. This is demonstrated by the 3D distribution of atoms shown in **Figure** **9**a, where Fe, Co, and Ni are uniformly distributed, but Cr and O are segregated. In addition, the O distribution also revealed that the native oxidation of the films occurs both on the surface and at the columnar grain‐boundary gap.

The isosurface of O and the curves of each elemental concentration distribution are exhibited in Figure 9b. Evidently, Cr segregates along the columnar grain boundaries with O and forms strip‐like shapes. The maximum Cr and O contents are approximately 30% and 13%, respectively. In the grain interiors, a uniform compositional distribution exists, and the O content is ≈3%. Therefore, as schematically illustrated in Figure 9e, a core–shell structure is formed, where the ferromagnetic Fe, Co, and Ni atoms form the core, and the Cr oxide acts as the shell layer (unlike the traditional spherical core–shell structures, the oxide shell analyzed in this study was wrapped around the exteriors of the columnar crystals). The Cr oxidation produces two significant effects. On the one hand, the Cr oxidation decreases the number of Cr—Cr pairs but increases the number of Cr—O—Cr pairs, which weaken the antiferromagnetic direct cation–cation interactions through the indirect cation–anion–cation interactions. Moreover, Goodenough et al. indicated that both 90° and 135° Cr—O—Cr pairs interactions are ferromagnetic,^[^ ^69^ ^]^ which was also confirmed by Zhang et al.^[^ ^70^ ^]^ Thus, the existence of Cr—O—Cr pairs contributes to the enhancement of ferromagnetism. On the other hand, the concentrations of Fe, Co, and Ni in the columnar crystal increase when an oxide layer is formed by the segregation of the antiferromagnetic Cr atoms at the columnar crystal boundary; this can strengthen the ferromagnetic exchange interaction, and thus, enhance the ferromagnetism.

##### Contribution of a Small Amount of Body‐Centered Cubic Phase that Exhibits Ferromagnetic Behavior

The CoCrFeNi_thick_ film exhibits an FCC and BCC dual‐phase microstructure. Although the ferromagnetic BCC content is small, it also contributes to the ferromagnetism of the film.

The aforementioned contents have reveals that there exist three factors that affect ferromagnetic behavior of HEATFs. But which are the main factors? First, all the HEATFs exhibit severe lattice distortion, which changes the exchange interaction distance and then enables FCC phase ferromagnetic at RT. Thus, it plays a critical role in terms of ferromagnetism. For the remained two factors, the core–shell structure and the BCC phase, the difference of the effect between them will be unveiled as follows.

**Figure 10:**
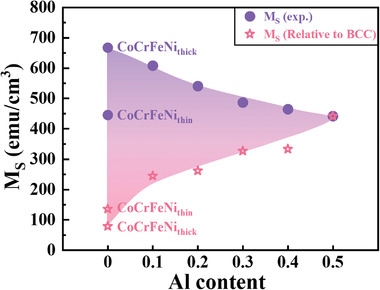
Comparison of the experimental *M*_S_ and relative *M*_S_ contributed by the BCC phase.

With the increasing Al content in the Al*_x_* CoCrFeNi films, the amount of the BCC phase increases, as shown in Figure 3c, which theoretically increases the ferromagnetism. If the BCC phase is considered to be the sole ferromagnetic sources of the films, based on the Al_0.5_CoCrFeNi film with the most BCC content, then the relative *M*_S_ contributed by the BCC phase for all the films can be inversely calculated as shown in **Figure** **10**. The relative *M*_S_ and experimental *M*_S_ show opposite trends. As the BCC phase content increases, the experimental *M*_S_ decreases gradually. The CoCrFeNi_thick_ film exhibits the lowest BCC content but the largest *M*_S_ value. Hence, we can conclude that the BCC phase contributes to the ferromagnetic behavior, but it is not the main source of ferromagnetism.

Because the difference between the electronegativity of Al and O (1.83) is larger than that of Cr and O (1.78),^[^ ^62^ ^]^ the native oxidation of Al in the Al*_x_* CoCrFeNi film occurs preferentially, as confirmed by the XPS analysis results shown in Figure 7. Moreover, Figure 9c,d show the 3D‐APT compositional distribution and the isosurface of Al (8 at%), as well as the concentration distribution curve of the Al_0.4_CoCrFeNi film. The addition of Al changes the core–shell structure into a composite structure with ferromagnetic Fe, Co, Ni and antiferromagnetic Cr as the core, and Al oxide as the shell. Therefore, the number of nearest‐neighbor Cr—Cr pairs increases in the columnar nanocrystal grains of the five‐element film containing Al, which reinforces the antiferromagnetic exchange interaction and then weakens the ferromagnetic properties with the increasing Al content (*µ*_i_ gradually decreases). This is consistent with the variations in the experimental *M*_S_. Therefore, the core–shell structure containing Al is detrimental to the magnetic properties.

The aforementioned analyses indicate that there are two most important factors to determine the ferromagnetic properties of the films: 1) Because of the severe lattice distortions in the HEATFs, the FCC phase exhibits ferromagnetism at RT, and 2) A core–shell structure, with Fe, Co, and Ni as the core and Cr oxide as the shell, exists in the HEATFs.

#### 4.1.2 Influence of Component Density on the Saturation Magnetization

A high *M*_S_ is necessary for soft magnetic materials, and it is closely related to the chemical composition density of the HEATFs. The magnetization (*M*) can be expressed by the following equation:^[^ ^38^, ^39^ ^]^

(5) $$M=\frac{n\mu_{H}^{2}H}{3KT}\;$$

where *n* is the total number of magnetic atoms per unit volume, *µ*_H_ is the atomic magnetic moment (*µ*_H,Fe_ = 2.2 *µ*_B_, *µ*_H,Co_ = 1.7 *µ*_B_, and *µ*_H,Ni_ = 0.6 *µ*_B_), *H* is the external magnetic field (all films can be saturated and magnetized when the external magnetic field reaches 15 kOe), *k* is the Boltzmann constant, and *T* is the thermodynamic temperature (the experiment was carried out at 298 K).

In the Al*_x_* CoCrFeNi films, the number of magnetic atoms (Fe, Co, and Ni) in a unit volume decreases with the increasing number of the non‐magnetic Al atoms. Hence, the *M*_S_ also decreases, as reported for bulk alloys and thin films. For instance, the *M*_S_ sharply reduced after the addition of non‐magnetic elements (such as Al, Si, or Cu) into the FeCoNi bulk alloys.^[^ ^28^, ^71^ ^]^ Additionally, for the series of CoFeNiCu films, the *M*_S_ of the film was almost the same for the same compositions.^[^ ^72^ ^]^

Although the CoCrFeNi_thin_ and CoCrFeNi_thick_ films have similar compositions, there are substantial microcracks in the former. These microcracks reduce the number of magnetic atoms per unit volume because of the lower total atomic density. Thus, the *M*_S_ of CoCrFeNi_thin_ decreases significantly. A similar conclusion can be obtained for a pure metal Co film.^[^ ^73^ ^]^

### 4.2 Low Coercivity Analysis

The coercivity increases when the domain wall movement is impeded by the microstructural discontinuities and sharp gradients.^[^ ^74^ ^]^ Therefore, the abundant microcracks in the CoCrFeNi_thin_ film would form a discontinuous interface for the magnetic flux, change the magnetic field distribution, and increase the leakage magnetic field above the cracks.^[^ ^75^ ^]^ Moreover, the microcracks can reinforce the pinning effect of the domain walls. Hence, the coercivity of the CoCrFeNi_thin_ film is larger than that of the CoCrFeNi_thick_ film. To eliminate the influence of the defects on the coercivity, we analyzed the relationship between the coercivity and the microstructure of the films without microcracks.

According to the technical magnetization theory,^[^ ^38^ ^]^ *H*_C_ is mainly related to the defect‐induced irreversible movement of the domain walls as well as the irreversible rotation of the magnetic moments, which are influenced by the magnetic anisotropy. Nanocrystalline soft magnetic materials exhibit a single domain structure owing to the small crystal grain sizes, and there is no domain‐wall movement during the magnetization‐reversal process. Therefore, the *H*_C_ is related to the nanoparticle size, as shown in Equation (6):^[^ ^76^ ^]^

(6) $$H_{c}=P_{c}\;\frac{\left\langle K_{1}\right\rangle }{J_{s}}\approx P_{c}\frac{K_{1}^{4}D_{p}^{6}}{J_{s}\;A{{}^{\prime }}^{3}}$$

where *P*_c_ is the dimensionless pre‐factor, *K*_1_ is the magneto‐crystalline anisotropy (according to the random anisotropy model, it is almost 0),^[^ ^77^ ^]^ *J*_s_ is the saturation polarization, *D*_p_ is the nanoparticle size, and *A*′ is the exchange stiffness constant between the nanocrystalline grains.

In our case, the average grain sizes of the HEATFs were 5–20 nm, and based on the above‐mentioned model, the calculated *H*_C_ was ≈1–15 A m^−1^. However, these calculated values are significantly different from the measured values for the films, and it is unable to explain the significant variations in *H*_C_ with small changes in the grain size either.

**Figure 11:**
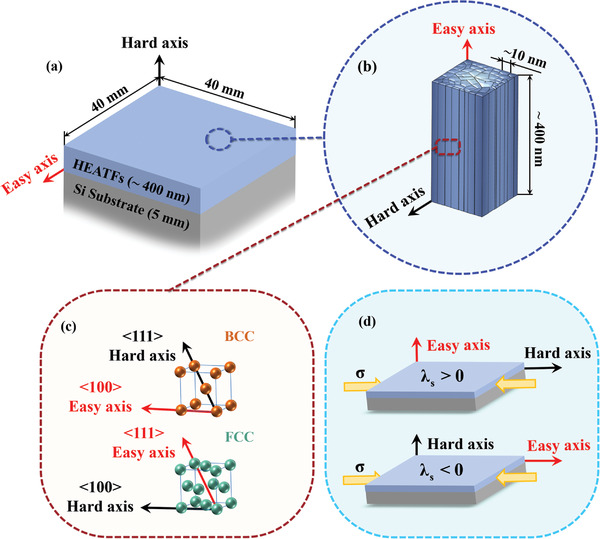
Schematic illustration of magnetic anisotropy of the HEATFs: a) Macroscopic shape anisotropy (*K*_m_). b) Columnar crystal‐shape anisotropy (*K*_s_). c) Magneto‐crystalline anisotropy (*K*_c_). d) Stress anisotropy (*K*_*σ*_).

The relation $H_{c}\propto D_{p}^{6}$ is an ideal model and only reflects the effects of the magneto‐crystalline anisotropy on *H*_C_; this is suitable for amorphous/nanocrystalline soft magnetic materials. However, the magnetic anisotropy of the HEATFs with columnar nanocrystals is complex and consists of the following four parts,^[^ ^78^, ^79^, ^80^, ^81^, ^82^ ^]^ as shown in **Figure** **11**.

a) Macroscopic shape anisotropy (*K*_m_) (Figure 11a): Distribution of magnetic dipoles leads to a large demagnetization field in the relatively‐small dimension (film normal direction), which is difficult to magnetize, and the opposite trend is observed in the relatively‐large dimension (film plane direction). The thickness of the films (400 nm) is much smaller than the planar size (40 mm). Therefore, the easy axis of magnetization is in the direction of film plane, and the hard axis is along the normal direction.
b) Columnar crystal anisotropy (*K*_s_) (Figure 11b): The average width of the crystal grains is ≈10 nm, and their height is ≈400 nm. Therefore, the direction along the length of the columnar crystal is the easy axis, while that of the width of the columnar crystal is the hard axis.
c) Magneto‐crystalline anisotropy (*K*_c_) (Figure 11c): The anisotropy of the atomic arrangement can induce a difference in the magnetization along the various crystal directions, and the easy or hard axis depends on the distance between the atoms in the crystal. For instance, the easy and hard axes of the BCC structure are <100> and <111>, respectively. By contrast, the easy and hard axis of the FCC structure are <111> and <100>, respectively.
d) Stress anisotropy (*K*_*σ*_) (Figure 11d): During the thin‐film growth, a residual stress can appear because of the differences in the crystal structures and coefficients of thermal expansion between the substrate and the film. Under stress, the easy axis must be in a direction parallel or perpendicular to the stress (related to the magnetostriction coefficient, *λ*_s_). For example, when *λ*_s_ > 0, the easy axis is perpendicular to the direction of compressive stress; in other case, it is along the direction of compressive stress.

The coercivity of the film will be affected if the magnitudes of the above‐mentioned magnetic anisotropy constants approach that of <*K*_1_> indicated in Equation (6).^[^ ^83^ ^]^ Hence, the complex magnetic anisotropy constants of the HEATFs are combined into an effective anisotropy constant, *K*_eff_:^[^ ^84^, ^85^ ^]^

(7) $$K_{\mathrm{eff}}=K_{m}+K_{\sigma }+K_{c}-K_{s}$$

The coercivity of the soft magnetic HEATFs is related to *K*_eff_ and *M*_S_ as:^[^ ^86^, ^87^ ^]^

(8) $$H_{C}=pK_{\mathrm{eff}}/\mu_{0}M_{S}=pE_{A}/\mu_{0}M_{S}\sin^{2}\theta$$

where *p* is a dimensionless factor, which depends on the specific type of magnetization; *µ*_0_ is the vacuum permeability (constant); *E*_A_ is the difference in the magnetic anisotropy energy per unit volume of the film along the in‐plane direction and that perpendicular to the film (it is the difference between the areas enclosed by the in‐plane and out‐of‐plane magnetization curves and the axis); and *θ* is the angle between the anisotropic easy axis and saturation magnetization, *M*_S_.

**Figure 12:**
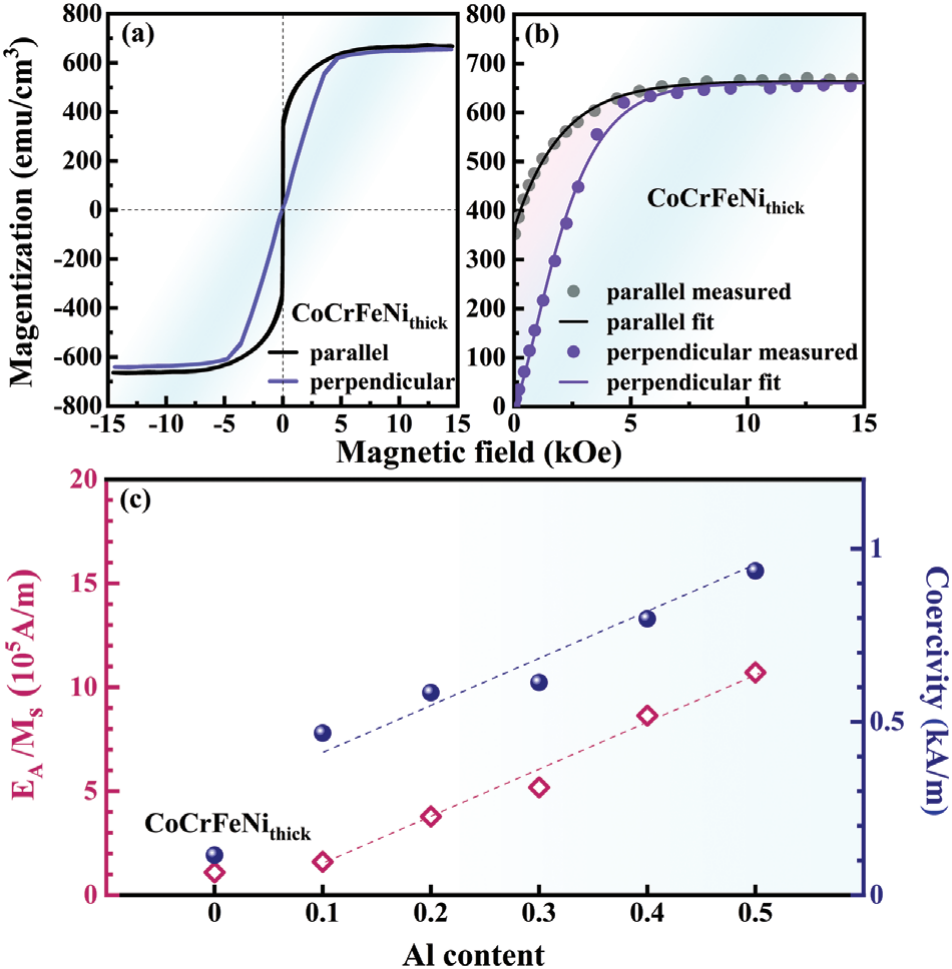
a) Hysteresis loops of CoCrFeNi_thick_ film along the directions parallel and perpendicular to the planes. b) The average magnetization curve and its fitting result. c) The values of *E*_A_/*M*_S_ and *H*_C_ for the CoCrFeNi_thick_ and Al*_x_* CoCrFeNi (*x* = 0.1–0.5) films.

The microstructures of all the Al*_x_* CoCrFeNi films exhibit FCC and BCC dual‐phases, and their grain sizes, thicknesses, and stress states show negligible changes. In this case, the films have nearly the same dimensionless factors, *p*. Moreover, the *µ*_0_, *p*, and *θ* in Equation (8) can be considered as constants. Hence, the coercivity is mainly related to *E*_A_/*M*_S_. As an example, the measured hysteresis loops of the CoCrFeNi_thick_ film along the directions parallel and perpendicular to the planes are shown in **Figure** **12**a. The average magnetization curve is used for fitting, as indicated in Figure 12b (the pink shaded area is *E*_A_). The fitting and calculation results for the other films are presented in Figures S6–S13 and Table S2, Supporting Information. Figure 12c displays the values of *E*_A_/*M*_S_ and *H*_C_ for the CoCrFeNi_thick_ and Al*_x_* CoCrFeNi (*x* = 0.1–0.5) films. These parameters increase linearly with the increasing Al content, indicating that the coercivity is mainly related to *E*_A_/*M*_S_. However, compared with the Al*_x_* CoCrFeNi films, the *E*_A_/*M*_S_ and *H*_C_ of the CoCrFeNi_thick_ film show significant deviations from the linear relationship owing to the different thicknesses of these films and the dimensionless factor, *p*. Furthermore, *H*_C_ of the CoCrFeNi_thick_ film is smaller, primarily because its magnetic domain is significantly smaller than that of the other films as shown in Figure 6.

## 5 Advantages of the Core–Shell Structure for the Magnetic Properties

According to the aforementioned results, the *M*_S_ of the ferromagnetic CoCrFeNi‐based HEATF is mainly related to the ferromagnetic FCC phase at RT due to the severe lattice distortion and formation of a core–shell structure. The novelty of these results is that the CoCrFeNi‐based high‐entropy film can be considered as a soft magnetic composite material composed of a ferromagnetic FeCoNi matrix and a weak‐magnetic and high‐resistance Cr oxide coating layer. This core–shell structure possesses the advantages of traditional soft magnetic metals and ferrite materials as well as exhibits an increased electrical resistivity and reduced the eddy‐current losses.^[^ ^88^ ^]^ For example, the Fe powders with Fe_3_O_4_ and Fe_2_O_3_ coating layers prepared in situ using H_2_O and O_2_ to oxidize the surface exhibited an increased *M*_S_ from 193.5 to 214.1 emu g^−1^, an increased permeability from 64.1 to 88.3 at 50 mT and 100 kHz, and a reduced core loss from 819 to 691 mW cm^−3^ at 100 kHz.^[^ ^89^ ^]^ More significantly, in our case, the core–shell structure is formed naturally, which greatly simplifies the preparation process and indicates excellent application prospects.

Considering that the remarkable magnetic properties of the films originate from the unique structure in the native oxidation state, a novel idea can be proposed, that is, whether the films can achieve optimized composite effects with the addition of O. In the present work, the CoCrFeNiO_0.4_ and CoCrFeNiO_0.8_ films were prepared to validate, which exhibited excellent soft magnetic properties and electrical resistivities (CoCrFeNiO_0.4_:*M*_S_ = 535 emu cm^−3^, *H*_C_ = 23 A m^−1^, and *ρ* = 237 µΩ·cm; CoCrFeNiO_0.8_:*M*_S_ = 816 emu cm^−3^, *H*_C_ = 223 A m^−1^, and *ρ* = 348 µΩ·cm).

**Figure 13:**
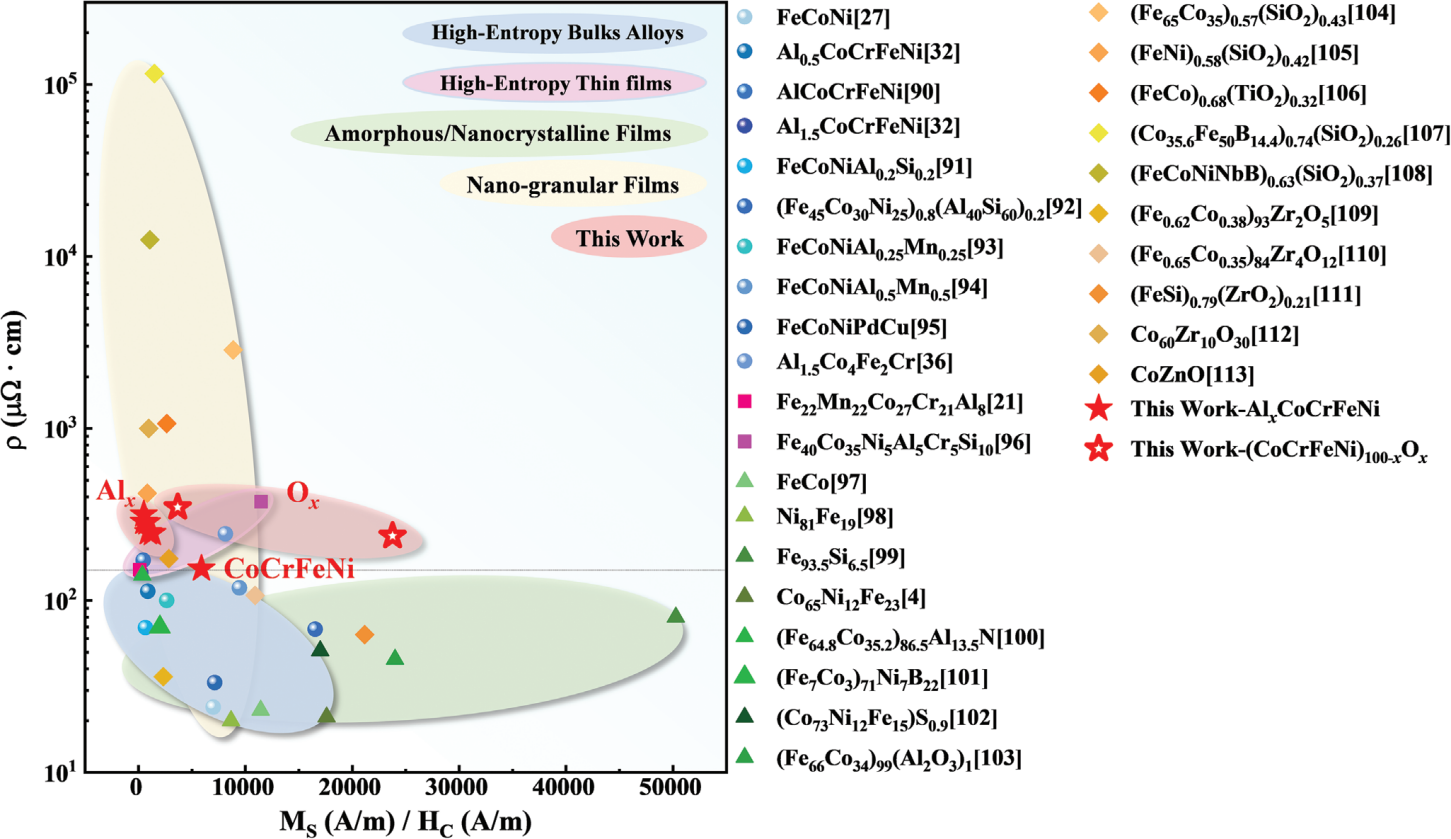
Soft magnetic‐performance parameters (*M*_S_/*H*_C_)–electrical resistivity (*ρ*) profiles of the investigated CoCrFeNi‐based HEATFs compared to those of bulk HEAs, other HEATFs and transitional thin films.^[^ ^4^, ^21^, ^27^, ^32^, ^36^, ^90^, ^91^, ^92^, ^93^, ^94^, ^95^, ^96^, ^97^, ^98^, ^99^, ^100^, ^101^, ^102^, ^103^, ^104^, ^105^, ^106^, ^107^, ^108^, ^109^, ^110^, ^111^, ^112^, ^113^ ^]^

Essentially, ideal high‐frequency soft magnetic materials should possess high *ρ*, high *M*_S_, and low *H*_C_. These basic parameters were evaluated using **Figure** **13**, in which *M*_S_/*H*_C_ is plotted on the horizontal axis, and *ρ* is shown on the vertical axis. A larger the *M*_S_/*H*_C_ value indicates a higher functional efficiency as well as a lower hysteresis loss. Moreover, a higher the *ρ* results in lower the eddy‐current losses. The materials with preferable high‐frequency soft magnetic properties are located at the upper‐right corner of Figure 13. The properties of all the CoCrFeNi‐based films are compared with those of the reported soft magnetic bulk HEAs, HEATFs, and other traditional soft magnetic films, such as amorphous/nanocrystalline films and nano‐granular films (comparison only with materials that are characterized by *M*_S_, *H*_C_, and *ρ*).^[^ ^4^, ^21^, ^27^, ^32^, ^36^, ^90^, ^91^, ^92^, ^93^, ^94^, ^95^, ^96^, ^97^, ^98^, ^99^, ^100^, ^101^, ^102^, ^103^, ^104^, ^105^, ^106^, ^107^, ^108^, ^109^, ^110^, ^111^, ^112^, ^113^ ^]^ A material with *ρ* > 150 µΩ·cm can be considered as a high‐resistance material (above the black‐dotted line in the figure). The electrical resistivities of the CoCrFeNi‐based HEATFs are higher than 150 µΩ·cm, indicating that these electrical resistivities are twice that of the bulk HEAs and amorphous/nanocrystalline films. For nano‐granular films, although the *ρ* can be as high as 10^5^ µΩ·cm, *M*_S_/*H*_C_ is mostly less than 5000 because of the large number of non‐magnetic semiconductor oxides. In contrast, the CoCrFeNi‐based HEATFs prepared in this study showed the most favorable high‐frequency soft magnetic properties.

The addition of Al can appropriately increase *ρ*, but slightly reduce *M*_S_/*H*_C_. The addition of O can increase both *ρ* and *M*_S_/*H*_C_, which significantly expands the performance modulation range. The findings of this study showed that the soft magnetic properties of CoCrFeNi‐based HEATFs can be adjusted in a wide range. A detailed study on the evaluation of the effect of O addition on the soft magnetic properties is ongoing, and the corresponding results will be reported in the future.

## 6 Conclusions

In the present study, soft magnetic CoCrFeNi thin film with FCC and BCC dual‐phases was prepared and the source of its magnetic properties had been systematically analyzed. The films showed a native oxidation‐produced core–shell structure, which comprised a core formed by the ferromagnetic elements Fe, Co, and Ni and a shell layer formed by the Cr oxide. As a result, the CoCrFeNi HEATFs exhibited both excellent soft magnetic properties and high electrical resistivities. The ferromagnetism behavior of the CoCrFeNi HEATFs, containing a large amount of FCC phase, are determined by the following: 1) Reinforced ferromagnetic exchange interaction due to the larger lattice distortion; 2) Weakened antiferromagnetic exchange interaction due to preferential oxidation of Cr; and 3) Contribution of the small amount of the ferromagnetism BCC phase. For the CoCrFeNi‐based HEATFs, the coercivity was mainly associated with the complex magnetic anisotropy and domain wall sizes.

The preferential oxidation of Cr can promote the formation of the aforementioned core–shell structure, and adjust the content ratio between the phase with ferromagnetism and that with a high electrical resistivity and weak magnetism to obtain optimized soft magnetic properties. Deliberately introducing O into HEATFs can optimize soft magnetic properties, further confirming the crucial role of core–shell structure. The CoCrFeNiO_0.4_ film showed the best properties, with an electrical resistivity of 237 µΩ·cm, a saturation magnetization of 535 emu cm^−3^ and a coercivity of 23 A m^−1^. However, the addition of Al in the films changes the core–shell structure, and then affects the soft magnetic properties. The results obtained in this study comprehensively reveal the crucial factors that determine the soft magnetic properties and provide a promising design strategy for soft magnetic HEATFs.

## Conflict of Interest

The authors declare no conflict of interest.

## Supporting information

Supporting Information

## Data Availability

Research data are not shared.
