# Supplementary material for: Native Oxidation and Complex Magnetic Anisotropy‐Dominated Soft Magnetic CoCrFeNi‐Based High‐Entropy Alloy Thin Films
Source: Adv Sci (Weinh). 2022 Oct 6;9(33):2203139. doi: 10.1002/advs.202203139 (PMC9685441; doi:10.1002/advs.202203139)
Supplement: Supplementary file 1 — Supporting Information [file ADVS-9-2203139-s001.pdf]

ADVANCED SCIENCE
Open Access

Supporting Information

for *Adv. Sci.*, DOI 10.1002/advs.202203139

Native Oxidation and Complex Magnetic Anisotropy-Dominated Soft Magnetic CoCrFeNi-Based High-Entropy Alloy Thin Films

Junyi Zhang, Xiao Wang, Xiaona Li, Yuehong Zheng, Renwei Liu, Junhua Luan, Zengbao Jiao, Chuang Dong and Peter K. Liaw*

Supporting Information

Native oxidation and complex magnetic anisotropy-dominated soft magnetic CoCrFeNi-based high-entropy alloy thin films

Junyi Zhang [1], Xiao Wang [1], Xiaona Li [1,] *, Yuehong Zheng [2], Renwei Liu[3], J. H. Luan[4], Z. B. Jiao[5], Chuang Dong [1], &Peter K. Liaw [6]

[1]*Key Laboratory of Materials Modification by Laser, Ion and Electron Beams (Ministry of Education), School of Materials Science and Engineering, Dalian University of Technology, Dalian 116024, China.*

[2]*State Key Laboratory of Advanced Processing and Recycling of Nonferrous Metals, Lanzhou University of Technology, Lanzhou 730050, China.*

[3]*Shimadzu China Co. LTD, Shanghai 200233, China*

[4]*Department of Materials Science and Engineering, City University of Hong Kong, Hong Kong, China.*

[5]*Department of Mechanical Engineering, The Hong Kong Polytechnic University, Hong Kong, China.*

[6]*Department of Materials Science and Engineering, The University of Tennessee, Knoxville, Tennessee 37996, USA.*

* Corresponding author, E-mail address: lixiaona@dlut.edu.cn (Xiaona Li)

Figure

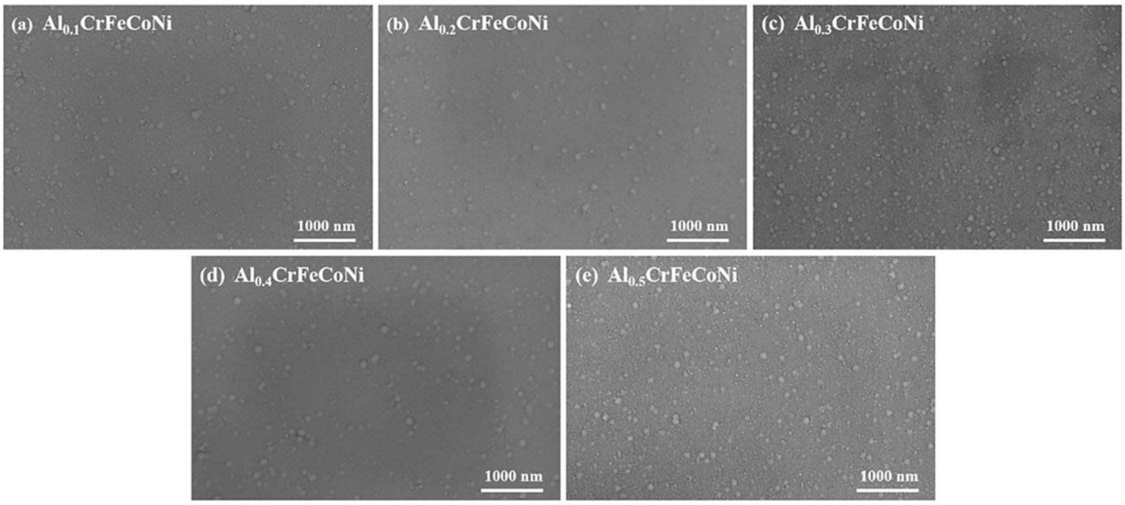

Figure S1. The SEM secondary-electron images of the in-plane surface morphologies of HEATFs.

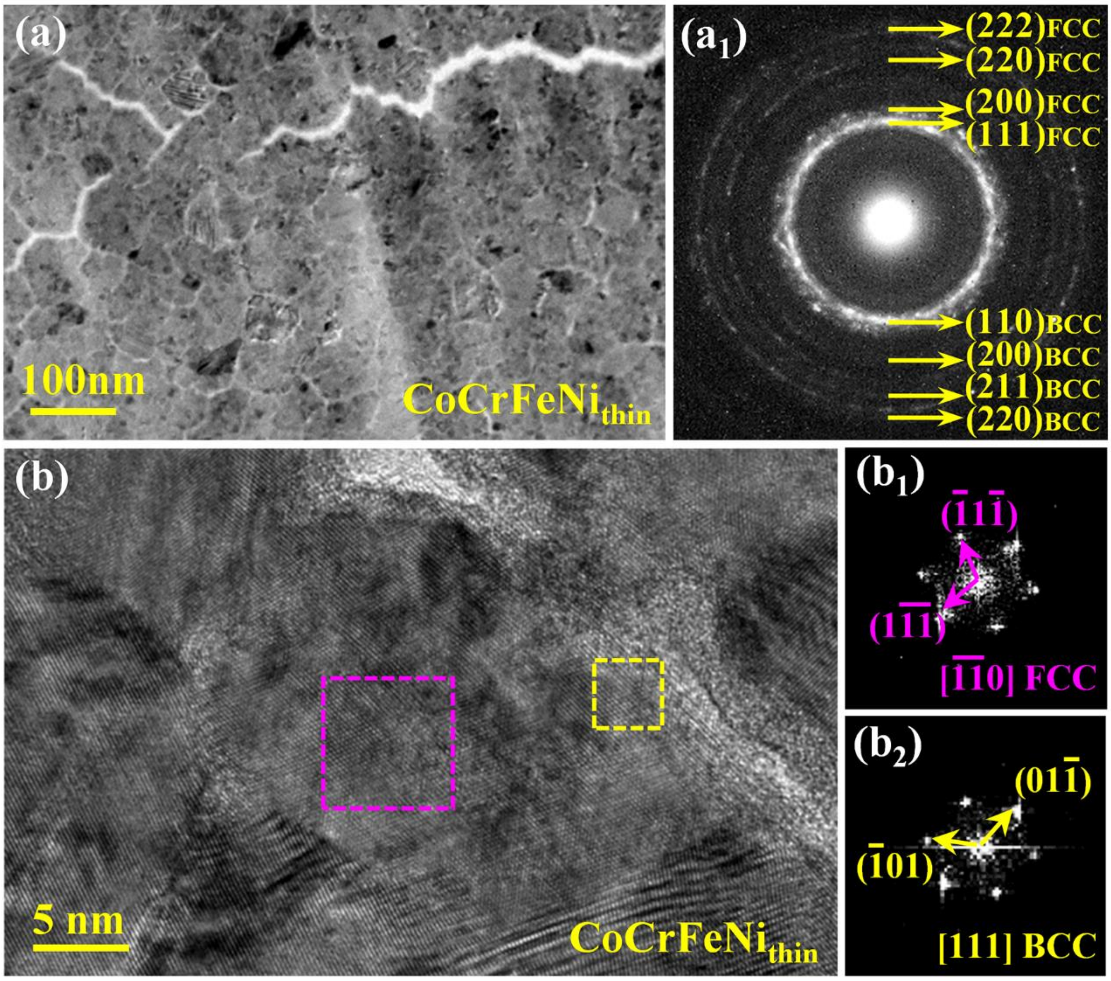

Figure S2. Microstructures of the $CoCrFeNi_{thin}$ film: the bright-field in-plane TEM morphology of $CoCrFeNi_{thin}$ film (a) and the (a_1)corresponding SAED pattern (b), the HRTEM images, the corresponding FFT spectra and filtered images of the FCC(b_1) and BCC(b_2) structures in the $CoCrFeNi_{thin}$ film.

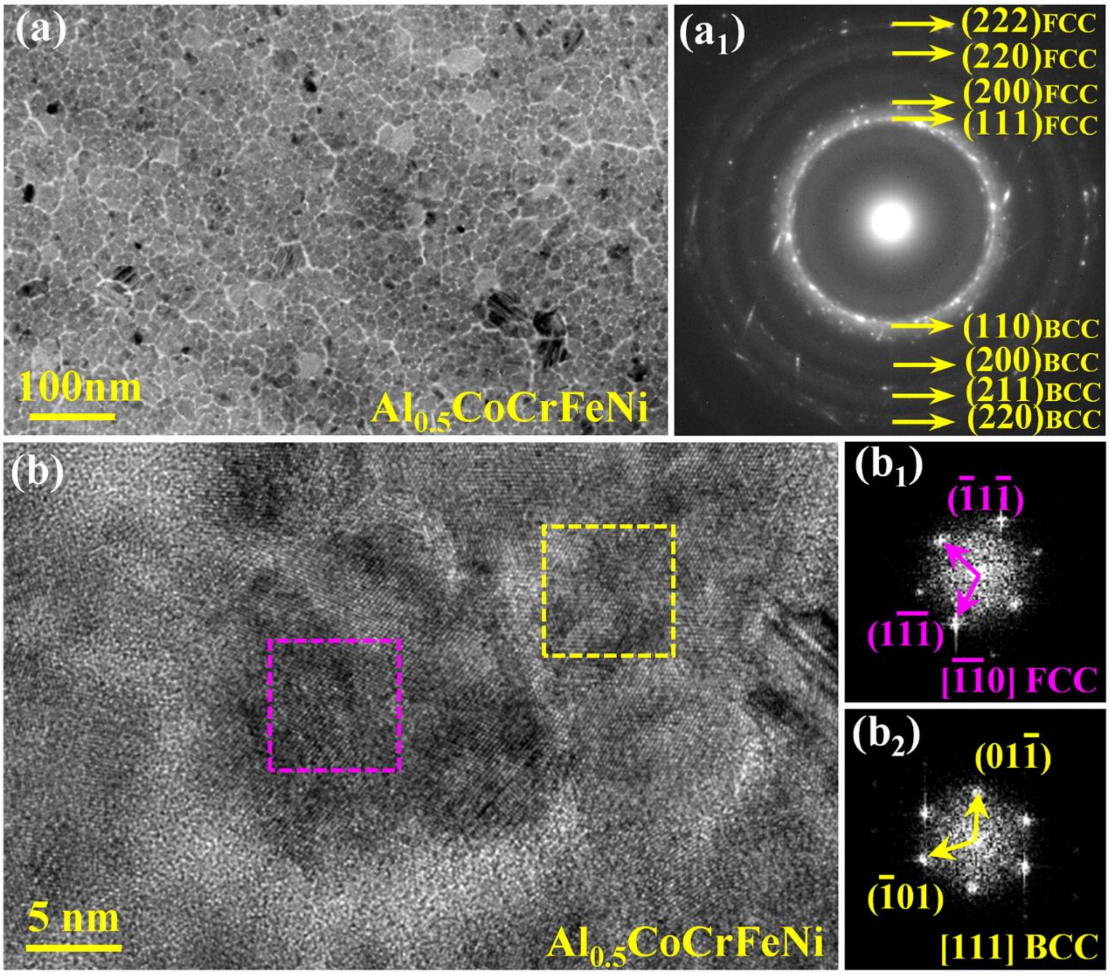

Figure S3. Microstructures of the $Al_{0.5}CoCrFeNi$ films: the bright-field in-plane TEM morphology (a) and the corresponding SAED pattern (a_1) The HRTEM images(b), the corresponding FFT spectra and filtered images of the FCC(b_1) and BCC(b_2) structures.

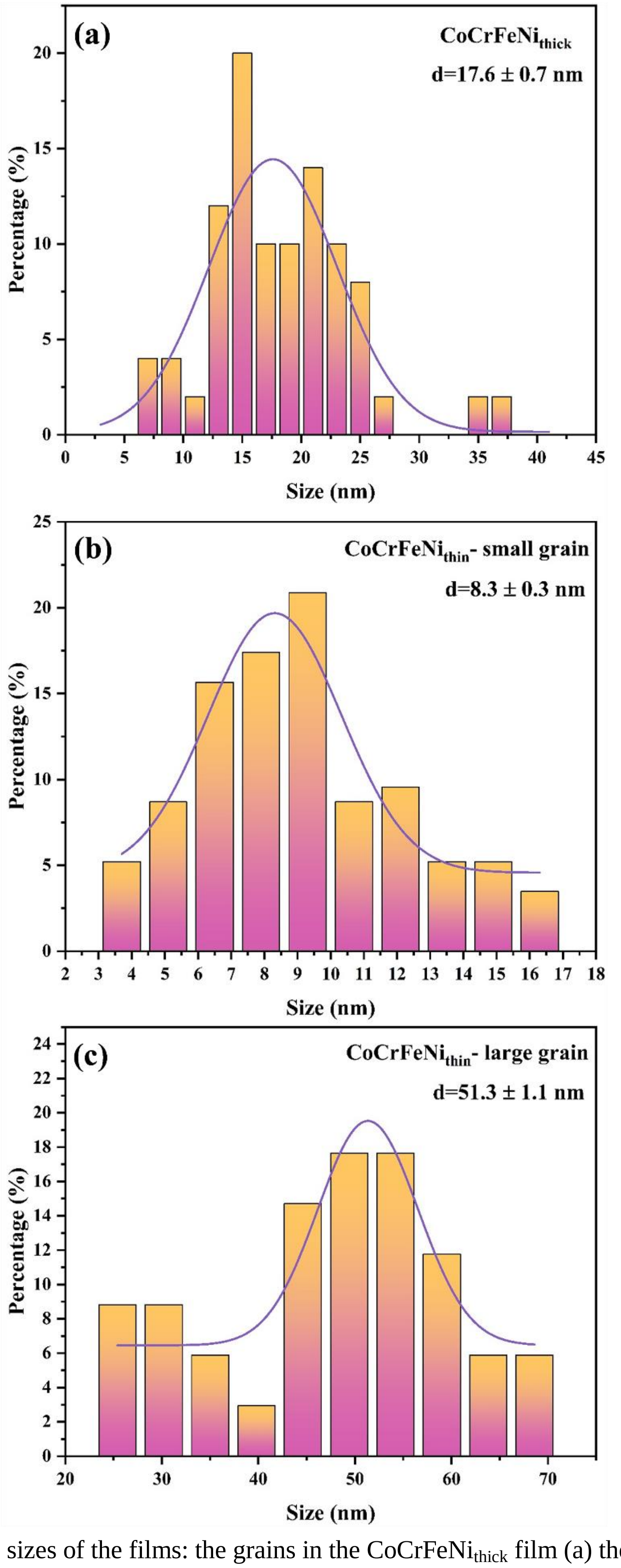

Figure S4. The grain sizes of the films: the grains in the $CoCrFeNi_{thick}$ film (a) the small grains in the $CoCrFeNi_{thin}$ film (b) the large grains in the $CoCrFeNi_{thin}$ film (c) (there are very few large grains, the statistical size value of large grains only accounted for 1/10 of the total grains statistical size value).

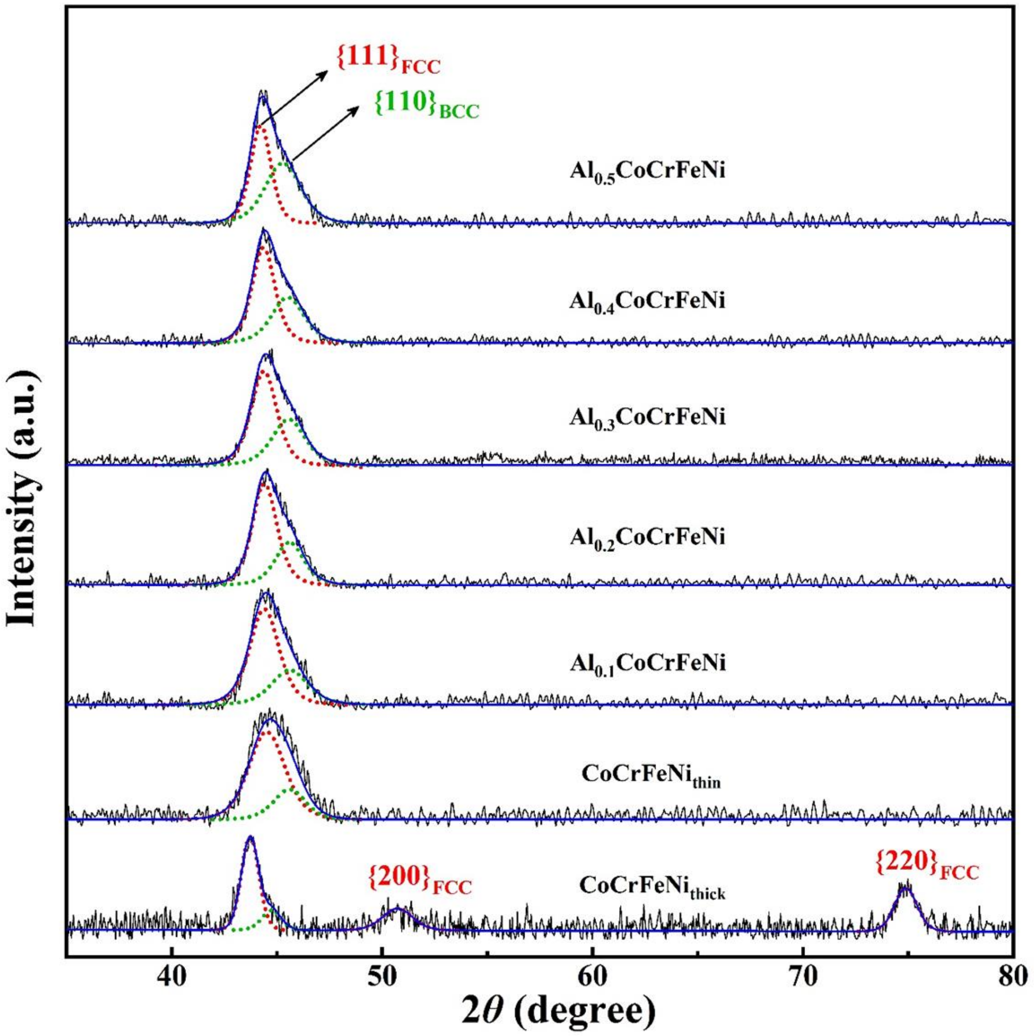

Figure S5. XRD fits patterns of the diffraction peak the as-deposited HEA films (red and green dash lines correlate to the diffraction peak of FCC and BCC, blue solid line is the sum of the fitting curves).

The content of the BCC phase

The α phase volume fraction (v_α) is related to the diffracted intensity of the X-ray beam on the {HKL} plane, ($I_{\{HKL\}\alpha}$), as follows:[1]

$$I_{\{HKL\}\alpha} = K \cdot R_\alpha \cdot v_\alpha \tag{S1}$$

where K is the constant for a measurement, R_α can be given by the following formula:[1]

$$R_\alpha = \frac{P_{\{HKL\}\alpha}}{V_\alpha^2} \frac{1+\cos^2 2\theta}{\sin^2\theta\cos\theta} \left|F_{\{HKL\}\alpha}\right|^2 A(\theta) e^{-2M} \tag{S2}$$

where $P_{\{HKL\}\alpha}$ is the multiplicity factor, $\frac{1+\cos^2 2\theta}{\sin^2\theta\cos\theta}$ is the Lorentz-polarization factor, $F_{\{HKL\}\alpha}$ is the structure factor of the {HKL} plane in the α phase, and V_α is the volume of the unit cell of α.

The present work, the relationship between the volume fractions of BCC and FCC phases in Al_xCoCrFeNi (x = 0 ~ 0.5) film is described as follows:

$$\begin{cases} \dfrac{v_{BCC}}{v_{FCC}} = \dfrac{I_{\{HKL\}BCC}}{I_{\{HKL\}FCC}} \cdot \dfrac{R_{FCC}}{R_{BCC}} \\ v_{BCC} + v_{FCC} = 1 \end{cases} \tag{S3}$$

Here, selecting $\{111\}_{FCC}$ and $\{110\}_{BCC}$ calculates the volume fraction. The amplitude squared of the structure factor ($\left|F_{\{HKL\}\alpha}\right|^2$) of FCC and BCC phases are $16f^2$ and $4f^2$, respectively, where f is the the atomic scattering factor for the FCC and BCC phases, which is closely related to the atomic number z.[1] The chemical disorder of high entropy films can ensure that the components doesn't fluctuate significantly in the FCC and BCC phases. Therefore, the effective atomic number of the two phases, Z_{eff}, are basically same.[2,3] Based on this feature, f is basically consistent in the two phases. **Table S1** shows the values of phase fractions and other parameters used for calculations.

Table S1. The values of phase fractions and other parameters

Sample	$P_{\{111\}_{FCC}}$	$P_{\{110\}_{BCC}}$	$\left(\frac{1+\cos^2 2\theta}{\sin^2\theta\cos\theta}\right)_{FCC}$	$\left(\frac{1+\cos^2 2\theta}{\sin^2\theta\cos\theta}\right)_{BCC}$	R_{FCC}/R_{BCC}	v_{BCC}
$CoCrFeNi_{thick}$			11.15	11.81	0.644	0.078
$CoCrFeNi_{thin}$			11.35	10.61	0.711	0.134
$Al_{0.1}$CoCrFeNi			11.45	10.78	0.721	0.242
$Al_{0.2}$CoCrFeNi	8	12	11.45	10.78	0.721	0.259
$Al_{0.3}$CoCrFeNi			11.46	10.81	0.722	0.324
$Al_{0.4}$CoCrFeNi			11.48	10.83	0.725	0.330
$Al_{0.5}$CoCrFeNi			11.55	10.95	0.729	0.438

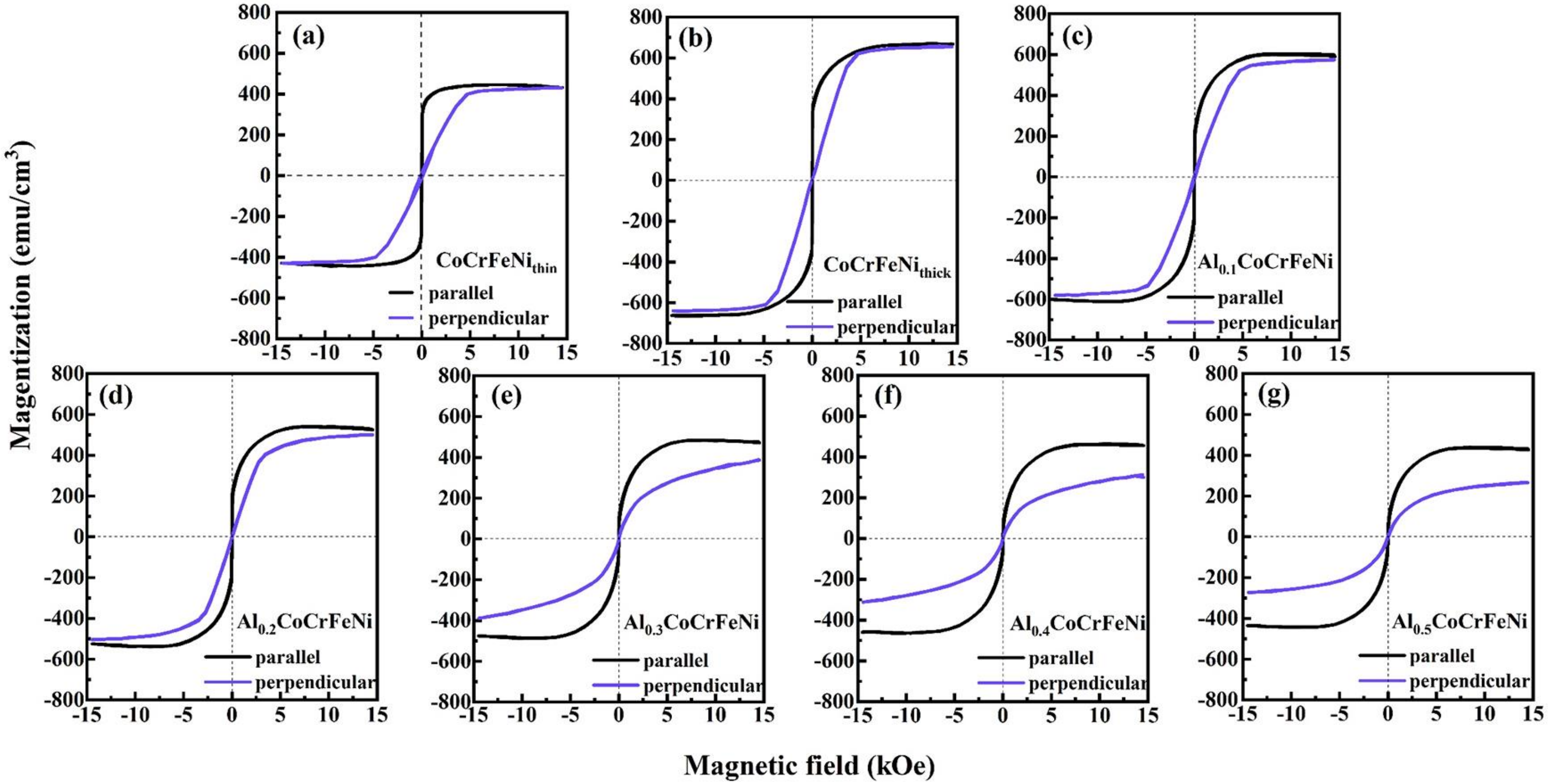

Figure S6. The in-plane and out-of-plane hysteresis loops of the as-deposited HEA films.

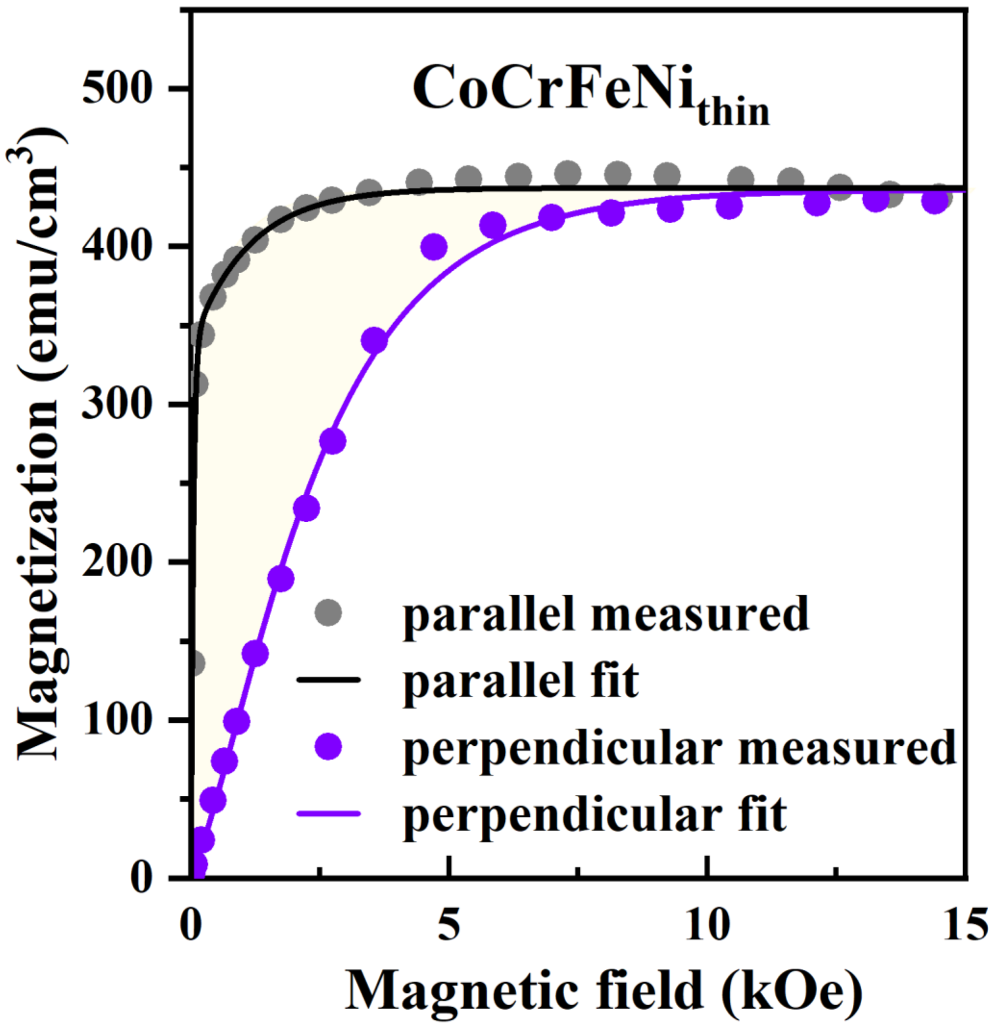

Figure S7. Appropriate curve fitting for magnetizing curves using the equation of $CoCrFeNi_{thin}$.

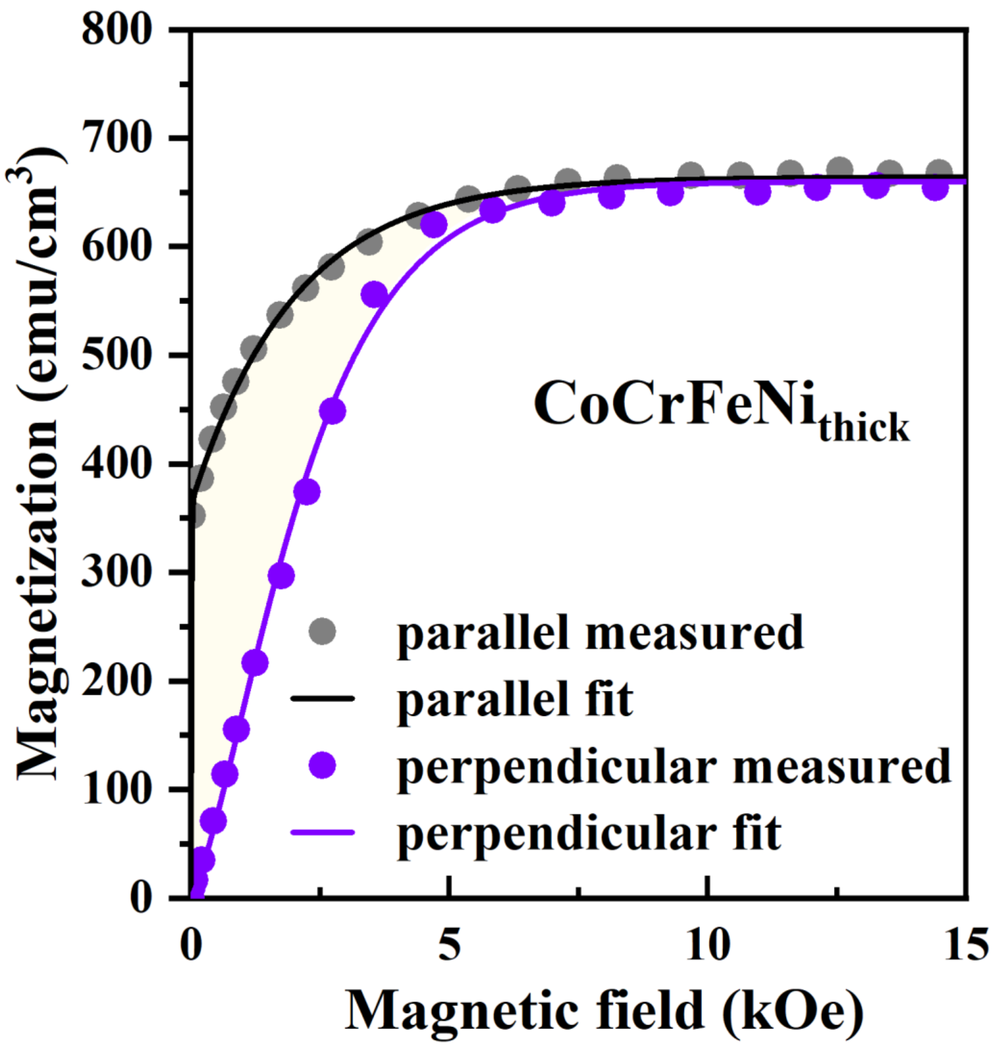

Figure S8. Appropriate curve fitting for magnetizing curves using the equation of $CoCrFeNi_{thick}$.

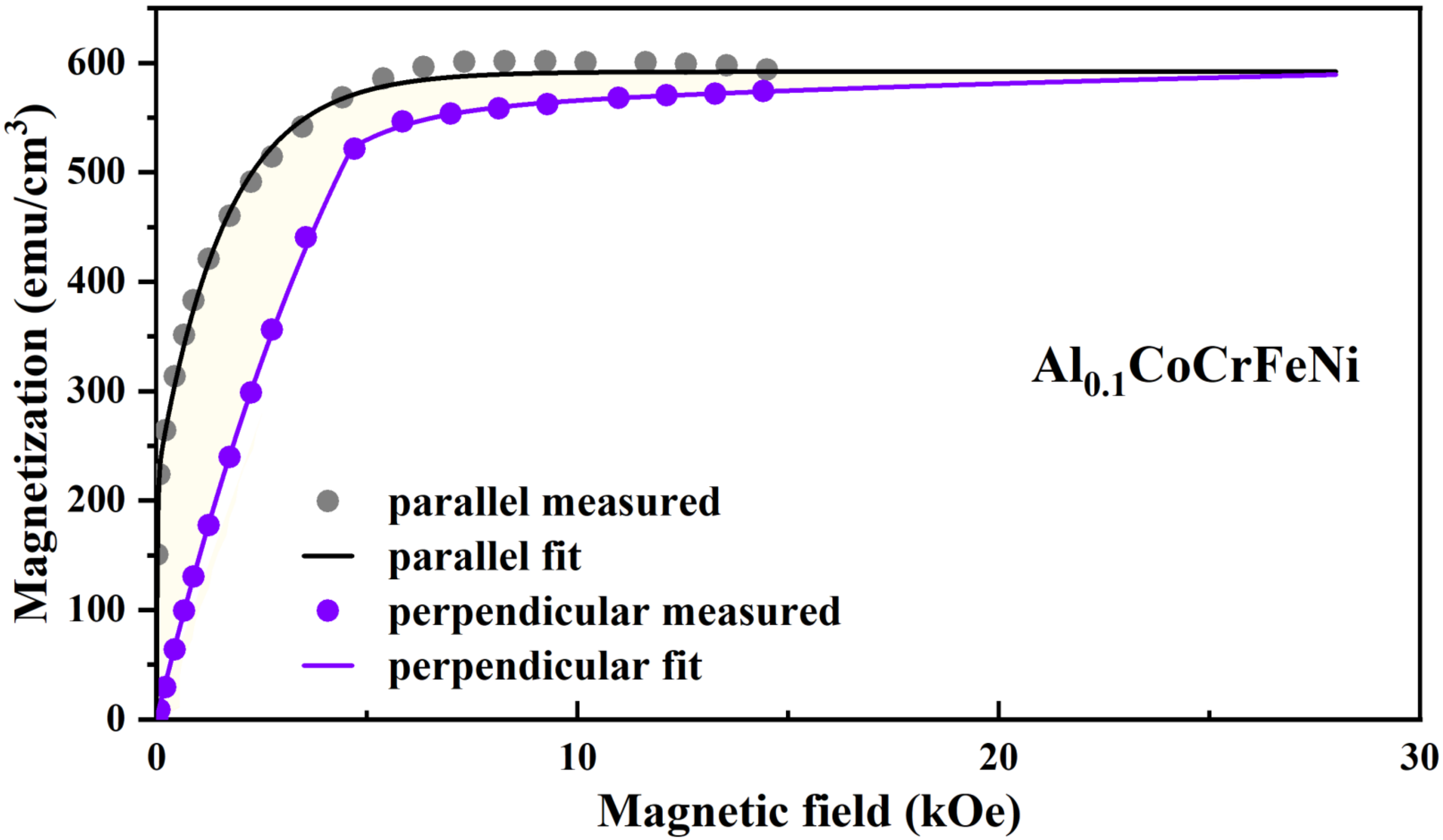

Figure S9. Appropriate curve fitting for magnetizing curves using the equation of $Al_{0.1}CoCrFeNi$.

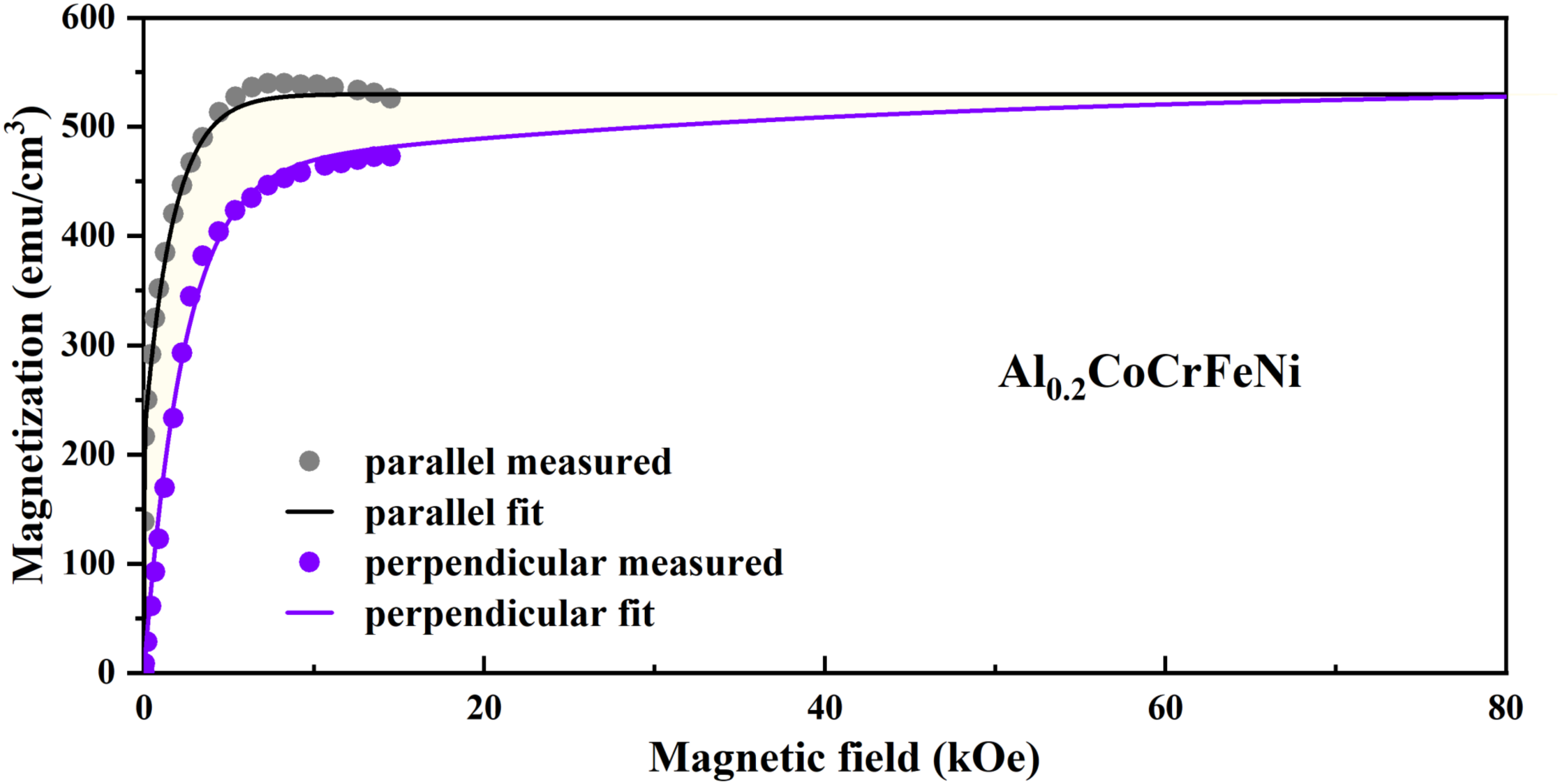

Figure S10. Appropriate curve fitting for magnetizing curves using the equation of $Al_{0.2}CoCrFeNi$.

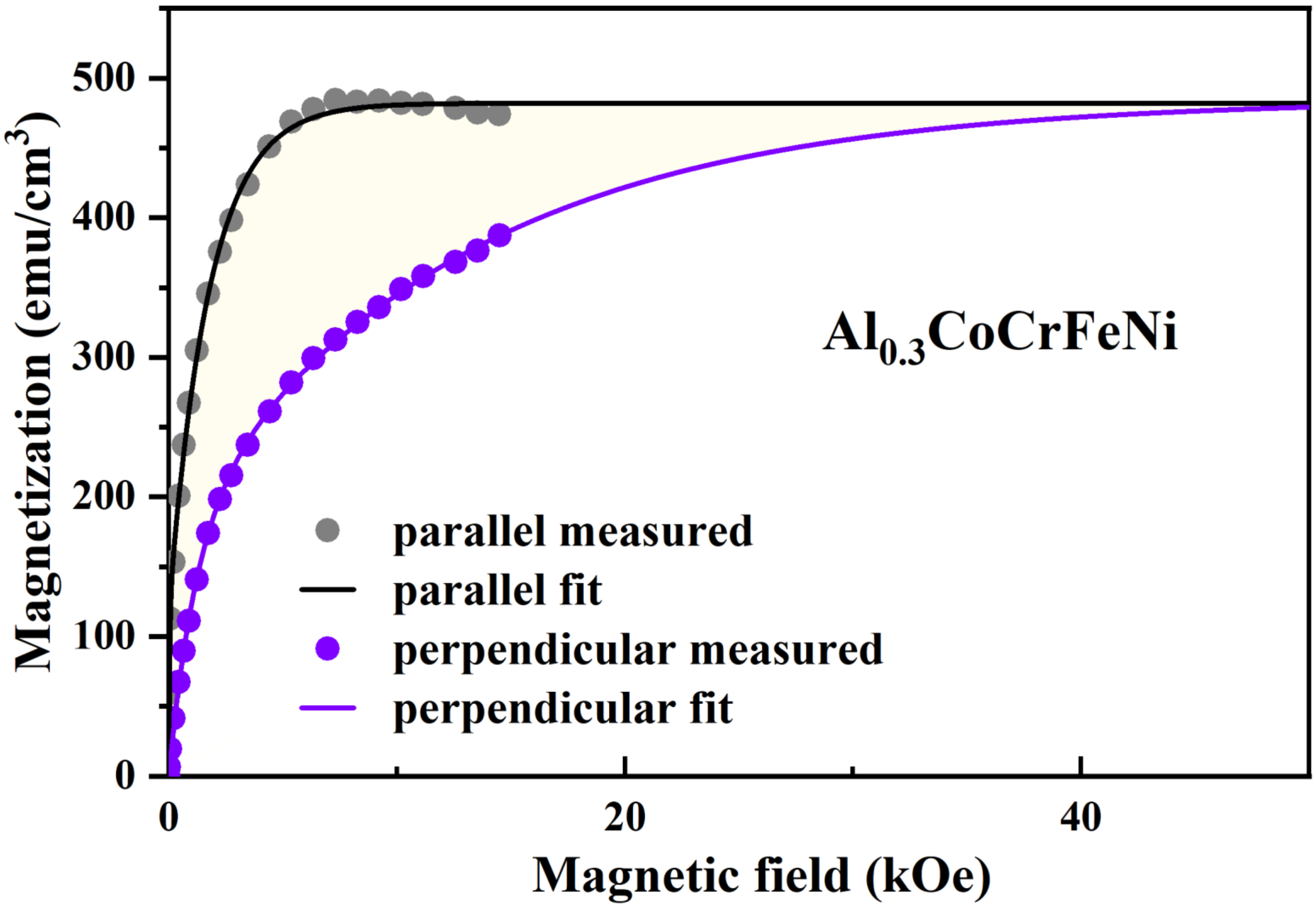

Figure S11. Appropriate curve fitting for magnetizing curves using the equation of $Al_{0.3}CoCrFeNi$.

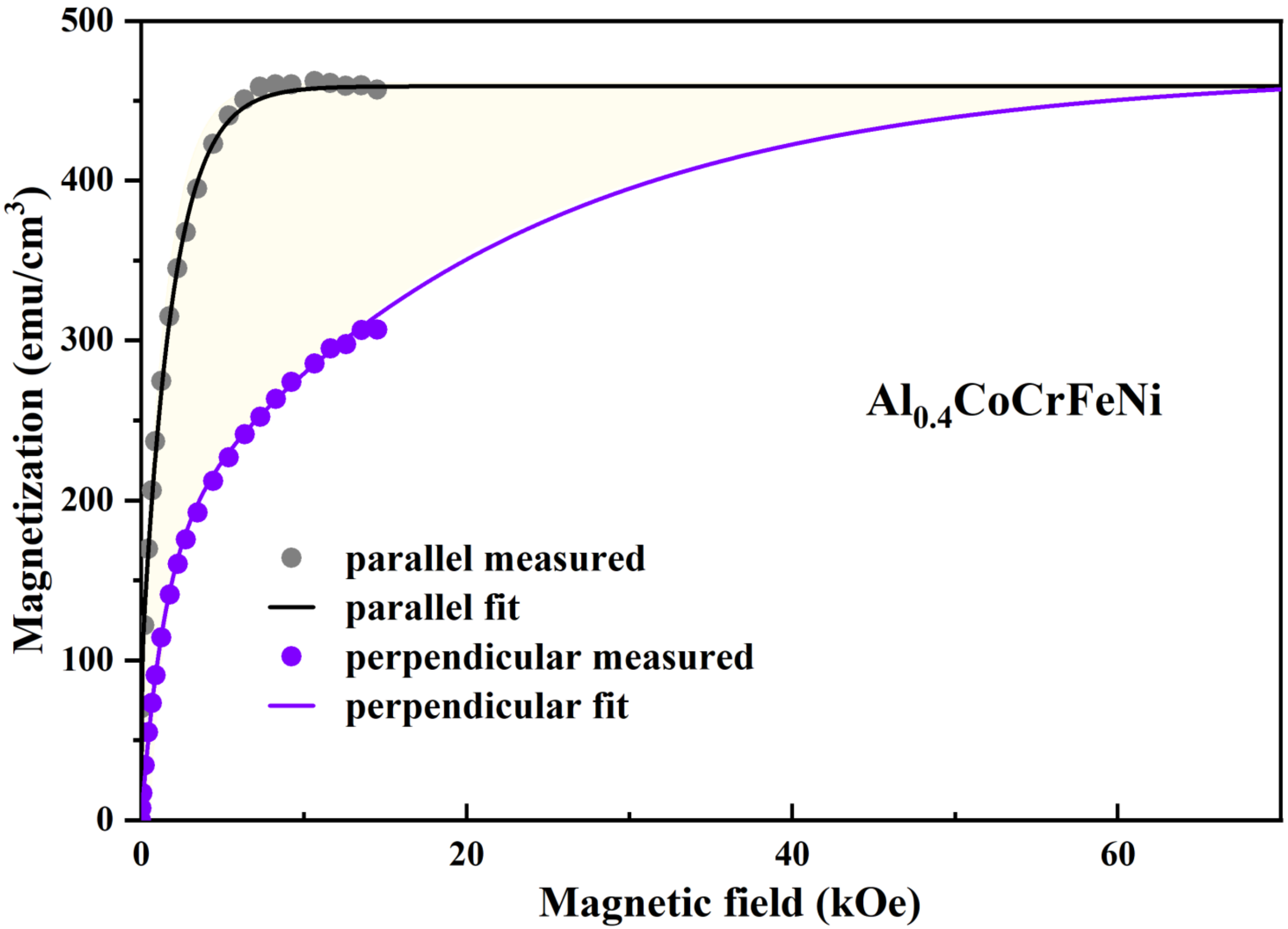

Figure S12. Appropriate curve fitting for magnetizing curves using the equation of $Al_{0.4}CoCrFeNi$.

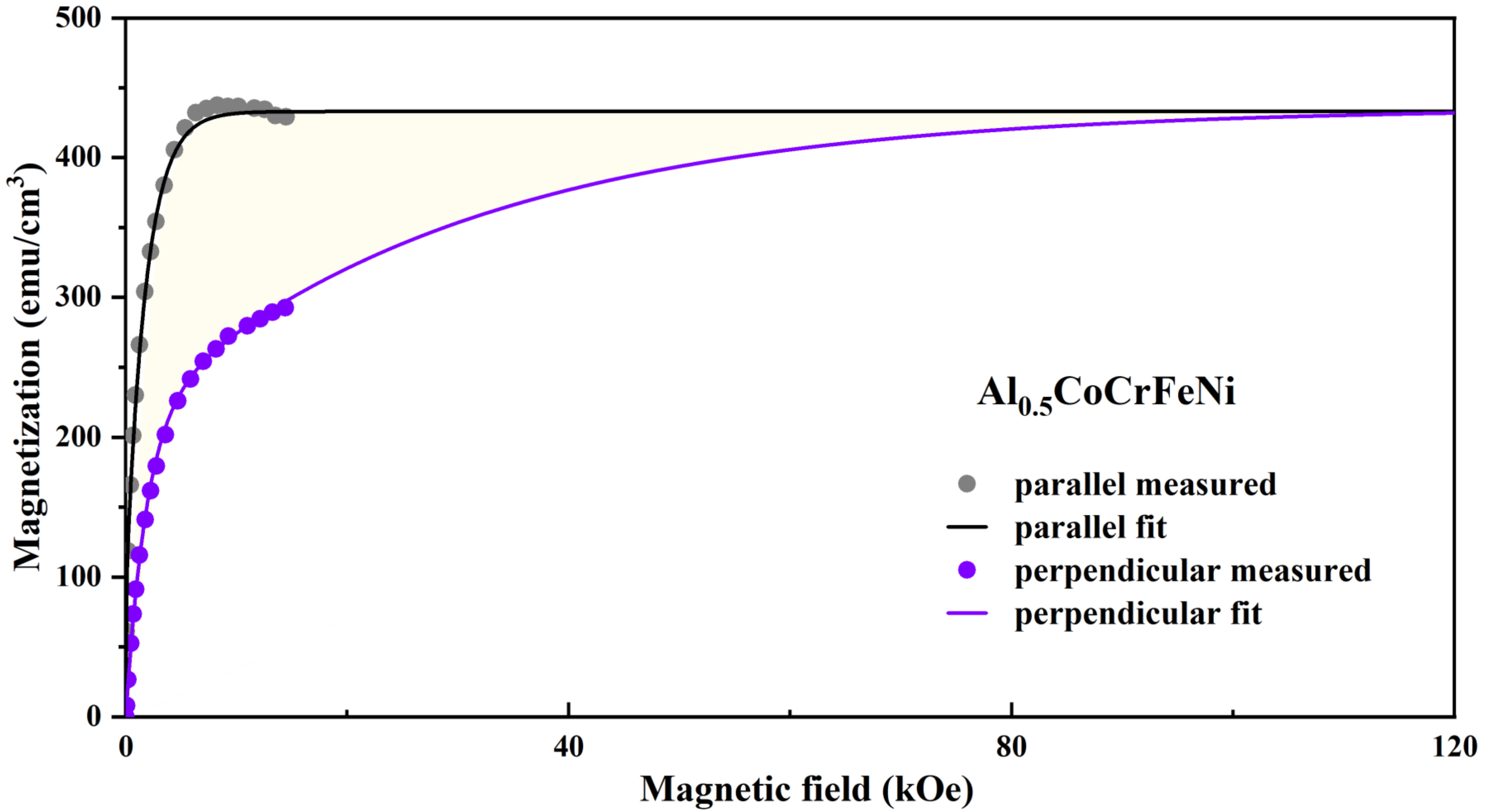

Figure S13. Appropriate curve fitting for magnetizing curves using the equation of $Al_{0.5}CoCrFeNi$.

Fitting Section

In soft magnetic materials, the area of the ring surrounded by the hysteresis loop is quite small, that is, the rising and falling stages of the curve are very close. We assume that each H value corresponds to only one *M*(*H*) value, so the M value in the magnetization curve can be obtained by the average value of M in the rising and falling stages of the hysteresis loop, as shown in the following equation:[4]

$$M_{\text{avg}} = \frac{M_u + M_e}{2} \tag{S4}$$

Where, M_u and M_e represent the magnetization of the upper and lower parts of the hysteresis loop in the first quadrant respectively, and it is assumed that they pass through the origin.

To obtain the effective anisotropy constant more accurately, it is often necessary to fit the magnetization curve to determine the in-plane magnetization direction and the integral area difference between the in-plane magnetization direction and the vertical axis. Therefore, in order to obtain better fitting effect, as shown in the following equation:

$$M = a[1-\exp(-bH)] + c[1-\exp(-d \text{[illegible]} \tag{S5}$$

The exponential term of this formula can produce a good fitting effect on the magnetization curve. By adding the two exponential terms, the fitting scope is wider and all our curve fitting parameters can be better fitted as follows:

Table S2. The fitting parameters and calculation for K_{eff} of the HEATFs.

Films	a		b		c		d		$\Delta E_A (\times 10^5)$
	Para	Perp	Para	Perp	Para	Perp	Para	Perp	
$CoCrFeNi_{thin}$	337.03	-204.76	2.26E-2	1.18E-3	99.97	640.76	8.99E-4	5.03E-4	9.83
$CoCrFeNi_{thick}$	298.64	-54405.16	4.71E-4	-8.05E-4	369.41	55073.16	9.13E-2	7.97E-4	8.56
$Al_{0.1}CoCrFeNi$	217.92	-171.20	4.429E-2	1.3E-3	374.08	763.20	6.16E-4	4.34E-4	10.07
$Al_{0.2}CoCrFeNi$	214.04	18.73	3.76E-2	1.53E-5	315.96	515.27	5.85E-4	3.68E-4	12.19
$Al_{0.3}CoCrFeNi$	120.02	310.80	2.55E-2	7.97E-5	362.04	174.20	5.62E-4	8.45E-4	31.68
$Al_{0.4}CoCrFeNi$	365.99	163.23	5.25E-4	7.31E-4	95.41	299.77	2.99E-2	4.80E-5	54.15
$Al_{0.5}CoCrFeNi$	92.18	306.00	2.08E-2	1.57E-5	343.90	134.00	5.54-4	8.46E-4	172.93

References:
[1] B. D. Cullity, *Elements of X-ray Diffraction*, Addison-Wesley, Reading, MA **1956**.
[2] R. C. Murty, *Nature* **1965**, *207*, 398.
[3] M. Vaidya, A. Prasad, A. Parakh, B. S. Murty, *Mater. Des.* **2017**, *126*, 37.
[4] F.C. Trutt, E.A. Erdelyi, R.E. Hopkins, *IEEE Trans. Power Appar. Syst.* 1**968**, *PAS-87*, 665.
